# The Role of Computed Tomography in Imaging Non-neurologic Disorders of the Head in Equine Patients

**DOI:** 10.3389/fvets.2022.798216

**Published:** 2022-03-07

**Authors:** Susanne M. Stieger-Vanegas, Ashley L. Hanna

**Affiliations:** ^1^Department of Clinical Sciences, Carlson College of Veterinary Medicine, Oregon State University, Corvallis, OR, United States; ^2^College of Veterinary Medicine, Washington State University, Pullman, WA, United States

**Keywords:** computed tomography, horse, head, paranasal sinus, teeth

## Abstract

Computed tomography (CT) imaging of the head in equine patients is now commonly performed as CT scanners are more readily available. Head CT has proven valuable in evaluating spatially complex anatomic structures, where radiographic superimposition, or restricted access *via* endoscopy or ultrasound, limit complete evaluation of the disease process. Head CT has been demonstrated to be incredibly valuable in the evaluation of dental and paranasal sinus disease, disease of the hyoid apparatus and ear, and in evaluation of skull trauma. CT is an excellent modality for assessment of both osseous and soft tissue structures; however, evaluation of complex vascular anatomy and determination of tissue viability is limited without the use of contrast agents. Therefore, various contrast agent protocols including intravenous and intraarterial iodinated contrast administration techniques have been established. CT also has limitations in the evaluation of brain and spinal cord disease, for which magnetic resonance imaging (MRI) has major advantages. Head CT images are most commonly evaluated in transverse planes. However, standard multiplanar reconstructions of the head including dorsal and parasagittal planes improve the understanding of spatially complex disease processes. These reconstructions can be crucial for accurate identification of diseased teeth and determination of the extent and severity of osseous and paranasal sinus disease. Head CT examinations are becoming an increasingly important diagnostic tool in the evaluation of horses with head disorders, and CT imaging findings are an important aspect in the clinical decision-making process. The following review discusses the indications, benefits, and technical considerations for the use of computed tomography (CT) in evaluating non-neurologic head pathologies in equine patients.

## Introduction

Diseases of the head of horses occur frequently and are often categorized by their origin. In a clinical setting, the first technique used to evaluate the head is often radiography or endoscopy. The most common reasons why equine head radiography is performed is to evaluate suspected dental or sinonasal disease or to evaluate injuries arising from a traumatic event (1). Head radiographs provide good spatial resolution; however, due to the superimposition and similarity in attenuation of the anatomic structures, localization of lesions can be quite challenging (2) and subtle lesions can even go undetected. In cases where clinical examination, radiography and endoscopy do not provide sufficient information or when there is incomplete or no response to treatment, additional diagnostic imaging modalities are often considered. The equine head is an anatomically and spatially very complex structure, and cross-sectional imaging techniques such as computed tomography (CT) or magnetic resonance tomography (MRI) are useful to eliminate superimposition of anatomic structures. While MRI is usually the preferred method for evaluation of the brain and spinal cord, CT is commonly the method of choice for evaluation of osseous or air-filled structures (3, 4). The purpose of this review is to provide an overview of the technical consideration and the CT appearance of common non-neurological conditions of the equine head.

## Technical Considerations

Clinical CT of the equine head has advanced rapidly over the last few years, including the advent of equipment that permits CT of standing sedated horses (5). Multidetector computed tomography (MDCT) units provide excellent resolution and allow scanning of the head in submillimeter slices (currently as low as 0.5 mm), providing outstanding detail of the osseous structures of the skull. Additional, isotropic voxel scanning is crucial to allow for distortion free reconstructions of the head in various planes. Furthermore, CT scanning of the head with a lower pitch value (pitch value <1) allows the use of a larger effective mAs when scanning, thereby creating similar to better image quality without necessarily increasing the radiation dose to the patient (6). The majority of these scanners require the horse to be fully anesthetized and recumbent during image acquisition. Complications associated with anesthesia and recovery from anesthesia are well described; in two studies it was demonstrated that over 80% of the anesthesia related complications occur in the recovery period (7) and despite improved anesthesia protocols, the mortality risk remains at ~0.9% in healthy horses (8). However, recent equipment and room modifications allow for the use of regular MDCT scanners in standing sedated horses. Additionally, specially designed cone beam CT units (CBCT) for imaging of standing horses are also available and use a pyramidal- or cone- shaped beam instead of the traditional fan- shaped beam of radiation in MDCT scanners (9). Strong sedation is needed for the CT examination in standing horses to reduce patient motion, and thereby reducing the risk of patient injury and equipment damage, avoiding general anesthesia make standing CT a lower risk procedure for the equine patient (10). Although these CBCT units are less expensive, these units have several limitations including those related to the size of the patient, motion of the standing sedated horse and high exposure settings needed to penetrate the high-density osseous structures of the head. The image quality is inferior to MDCT, especially for soft tissue structures (5); however, in one study assessing the agreement to detect abnormalities in equine cadaver heads, there was perfect agreement in detecting dental abnormalities including diastema, clinical crown and infundibular abnormalities but an only moderate agreement in identifying pulpar changes. Identification of pulpar changes on CBCT was unreliable due to the degree of scatter radiation, artifacts and the lack of Hounsfield calibration (11). The high exposure settings inherent to all types of computed tomography can pose a safety risk for personnel restraining sedated horses (5), especially if the handler remains next to the horse during the scan. However, scatter radiation, which is the highest radiation exposure risk for the handler, may be reduced by reducing the mAs settings; halving the mAs can reduce scatter radiation by half thereby reducing personnel exposure dose and persistently result in comparable image quality as demonstrated in an experimental cadaveric head study (12). The benefit of being able to scan standing horses without general anesthesia and its associated risks and complications and the lower costs of performing standing CT is clinically important (13) and future technical developments will likely make standing MDCT and CBCT more readily available for use in horses.

### Viewing of Equine Head CT Images

Most commonly, CT images of the head are acquired and viewed in transverse planes; however, additional sagittal and dorsal reconstructions as well as oblique reconstructions in non-traditional planes can be helpful for evaluating the teeth, paranasal sinuses, hyoid apparatus and other structures in the horse. In order to create oblique reconstructions without distortion, isotropic voxel scanning needs to be performed. Isotropic, thin collimated scanning can create a tremendous volume of data offering a wide range of possibilities including 3-dimensional reconstructions (14, 15). Additionally, the window settings should be adjusted to the anatomic area assessed, and a very wide window width (e.g., 3,000 for petrous bone) and high window level (e.g., 700 for petrous bone) setting should be chosen to assess high density structures such as the teeth and petrous bone (16). Furthermore, attenuation measurements may help distinguishing between fluid and soft tissue. However, it is important to remember that there is no significant difference in attenuation values between fluid in cystic structures and free fluid in the sinus (empyema fluid) and that the measurements can be very variable when high attenuation streaks from beam hardening artifacts are present (17).

### Contrast Application Protocols for Equine Head CT

Conventional CT without contrast agent administration provides sufficient information for evaluation of dental abnormalities or osseous trauma; however, evaluation of soft tissues or lesions of the neurocranium may be limited due to difficulty differentiating soft tissue attenuating structures from each other (18, 19). This becomes of particular importance in evaluation of the nasal cavity and paranasal sinuses when differentiation of tissue from non-viable material is essential to reach the correct diagnosis. To overcome this limitation, various contrast administration protocols and techniques have been developed. Currently, all contrast agents used for vascular enhancement in CT are iodine based. Both intravenous and intraarterial contrast injection protocols have been described for evaluation of the equine head using CT. Intravenous injection protocols are most commonly reported; however, timing of image capture, contrast agent dose, and injection rate vary considerably between institutions. Intra-arterial injection protocols are of interest because of their potential to decrease contrast agent volume, in turn reducing expense and injection time. Successful intra-arterial injection protocols *via* catheterization of the right common carotid artery have been described in a small number of horses (20, 21); however, future studies investigating this technique are needed to evaluate the safety and diagnostic benefits in horses with various forms of head disease. Currently, there is limited information about CT contrast agent protocols in standing horses; however, it is likely that several of the protocols reported for anesthetized recumbent horses could be adapted for CT scanning of standing horses (5).

Reactions to intravenous or intraarterial injection of iodinated contrast agents have been rarely described in veterinary patients (22, 23) and no anaphylactic responses to iodinated contrast agents have been described in veterinary patients (24). Only transient, self-resolving cutaneous and cardiovascular reactions to intra-arterial administration of iodinated contrast medium into a distal limb have been reported in horses (25).

### Common Artifacts Noted During Equine Head CT

**Figure 1:**
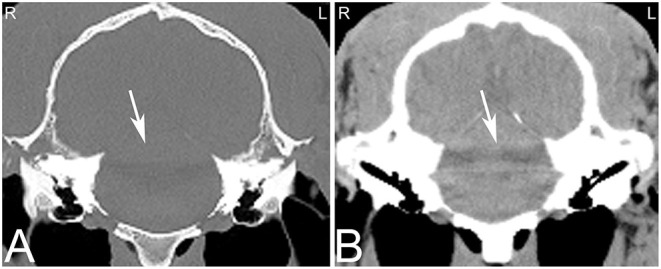
Beam hardening artifact. Transverse computed tomography images of the head at the level of the petrous bone in a bone **(A)** and soft tissue **(B)** window. The beam hardening artifact can be noted as hypoattenuating streaks (white arrows) next to areas of high attenuation such as the petrous bone of the skull. L, left; R, right.

A wide range of artifacts have been described in CT studies (26) and can pose problems in image evaluation. The most common artifacts in equine head CT are beam hardening, partial volume and streaking artifacts (5). If CT examinations are performed in standing sedated horses, motion artifacts are a common problem affecting image quality. In anesthetized horses, motion artifacts can arise around small vascular structures such as the intracranial or guttural pouch vessels where pulsation can lead to mis-registration and stair-step artifacts. While some of these artifacts are dependent on the imaging protocol used, others are intrinsic to the patient including beam hardening artifacts caused by surrounding bone (27) and streak artifacts caused by metallic objects such as metallic foreign bodies, implants, and dental fillings. Beam hardening artifacts appear as hypoattenuating streaks next to areas of high attenuation such as the petrosal part of the temporal bone (Figure 1) as a result of selective attenuation of low energy rays from the x-ray beam while high energy x-rays are less affected (i.e., “hardening of the x-ray beam”). Beam hardening artifacts can also be seen in the central aspect of the brain as a small area of hypoattenuation caused by the resorption of low-energy x-rays by the surrounding bones of the skull. Since the intracranial structures are surrounded by bone, this area is particularly prone to beam hardening artifacts. Streaking artifacts are also caused by selective attenuation of low energy x-rays and have similar image appearance and are caused by hyperattenuating substances such as metallic objects or a high dose of contrast medium in the examined area. Adjustment of contrast agent protocols can help reducing streaking artifacts caused by contrast agents and can include diluting the contrast agent, using a saline chaser or prolonging time to image capture to reduce contrast agent concentration in the area of interest.

## Imaging Specific Disorders of the Equine Head

### Dental Disease

Dental disease is extremely common in horses and of major clinical importance (28, 29). Moderate and severe dental disease is reported in 45.3 and 8.4% of working horses in two provinces in Egypt (30), and in the United Kingdom from 1962 to 1963 up to 10% of veterinary practice time was spent on dental-related work in horses (31). Dental disease has been ranked as the third most common medical problem seen by equine practioner in the United States of America (32). Dental disease alone or in combination with sinus disease can result in a wide range of clinical signs including pain and discomfort, halitosis, dropping of feed or slow eating, facial swelling, and ocular or nasal discharge. The initial diagnostic investigation of these cases usually includes radiographs. However, CT can have great value in horses with normal radiographic findings and suspicious clinical signs, and in those with incomplete resolution of clinical signs despite apparently appropriate medical or surgical treatment. CT is advantageous when on radiography it is unclear if there is any dental involvement or if more than one tooth is believed to be involved in the disease process (33, 34).

**Figure 2:**
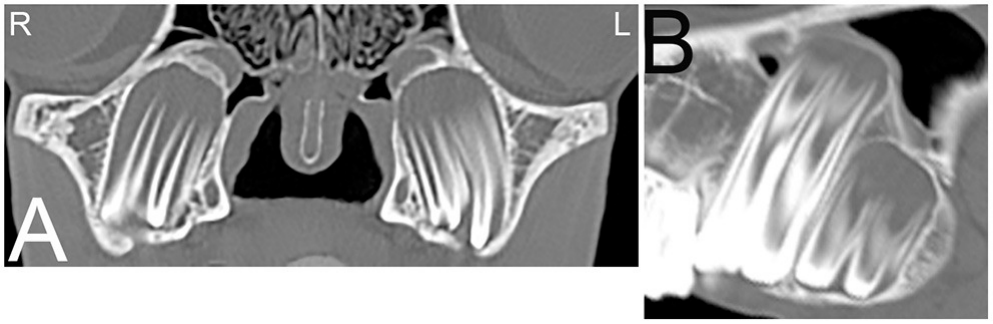
Eruption cysts. Transverse **(A)** and sagittal **(B)** computed tomography images of the head at the level of the third maxillary cheek teeth of a 1-year-old Miniature horse. Radiolucent eruption “cysts” are present adjacent to the immature apices of the maxillary cheek teeth and should not be confused with apical or periapical infections in young horses. L, left; R, right.

**Figure 3:**
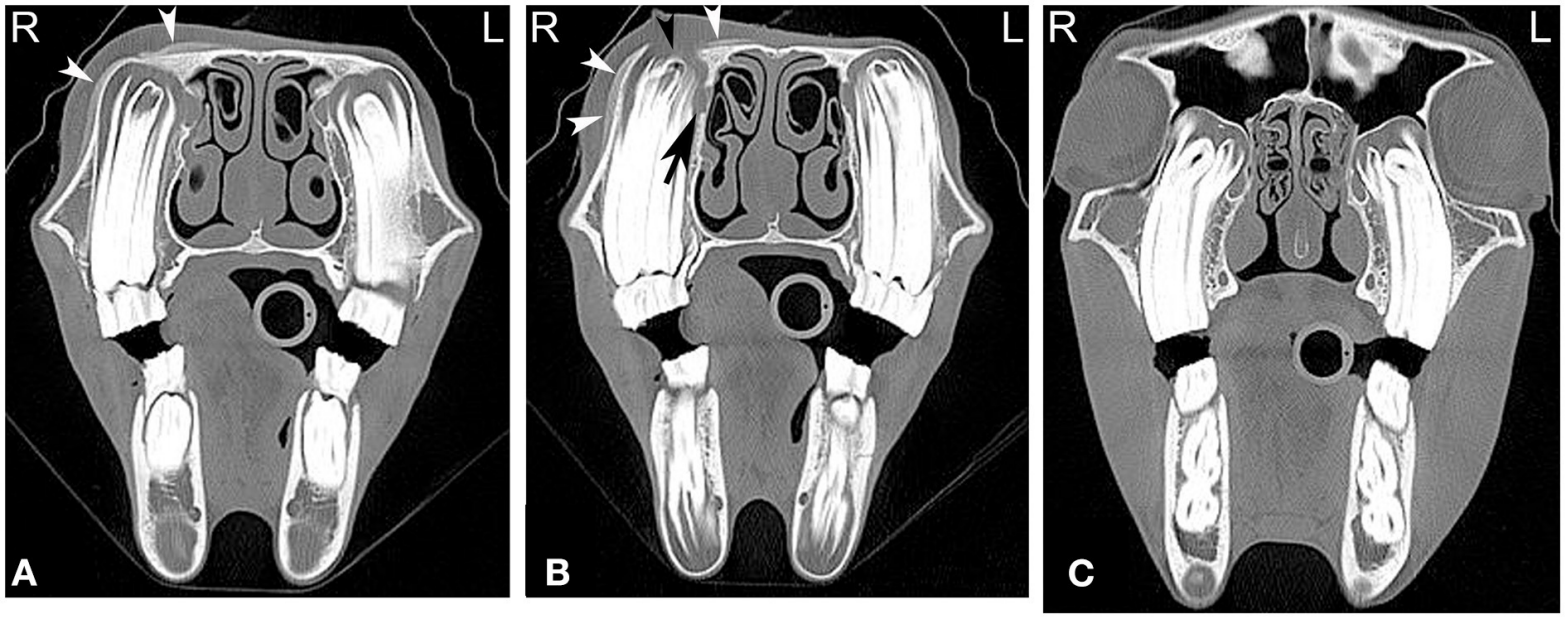
Maxillary soft tissue swelling with “caps” and sinusitis. Transverse computed tomography images of the head at the level of the premolar teeth displayed in a bone window of a 3-year and 11-month-old Miniature horse with a hard swelling rostral to the right eye. CT images at the level of the fourth premolar teeth **(A,B)** show deciduous cheek teeth remnants (“caps”) on all premolar teeth. Dorsal to the right fourth maxillary premolar tooth (Triadan 108), there is a focal area of increased soft tissue thickening with loss of the right maxillary bone (black arrowhead) dorsal to the apex of the right fourth maxillary premolar tooth (Triadan 108) and adjacent periosteal remodeling of the maxillary bone (white arrowheads). Additionally, widening of the periodontal space (black arrow), osteitis and small amounts of gas associated with the apex of this tooth are present, consistent with apical infection. CT image at the level of the third maxillary molar teeth **(C)**, whose alveoli extend into the caudal maxillary sinuses, decreasing the size of their lumina. L, left; R, right.

On radiographs or CT images, the developing tooth is first seen as an area of radiolucency close to the gum line within the mandible or maxilla. As the enamel of the tooth develops, the tooth becomes more visible. The developing enamel is folded, and the appearance of the unerupted tooth changes over time until its eruption with the appearance of a mature tooth (35). Up to the age of 2 years, young horses have up to 24 deciduous teeth including paired incisors (I) 3/3, canine teeth (C) 0/0, and premolar (P) 3/3 and molar (M) 0/0 teeth. The Triadan method is commonly used for identifying the teeth. On radiographic or CT images erupted deciduous incisor teeth are more radiolucent, have shorter reserve crowns and apices, and a shorter cross-sectional area than permanent teeth (36). Additionally, radiolucent, non-pathological “eruption cysts” can be present surrounding the immature apices of the teeth in young horses, which can be mistaken for apical infection (Figure 2). In young horses, the roots are not fully developed, and the term apex is commonly used. In young horses, the long reserve crown and apices of the cheek teeth lay deeply embedded in the bone of the mandible and maxilla and extend into the paranasal sinuses. The ventral aspect of the mandible can be irregular in shape due to deviation of the ventral mandibular cortex by the long reserve crown and apices of the cheek teeth. If these long reserve crowns and apices in young horses become infected, then infection of the adjacent bone and sinuses (Figure 3) can be noted (29).

The mature horse has 40–44 teeth (paired I 3/3, C 1/1 in the male or C 0/0 in the female, P 3/3 or 4/3 and M 3/3). All permanent incisor and cheek teeth in horses are hypsodont, continuing to erupt throughout life compensating for occlusal grinding ware of typically 2–3 mm/year. Due to this continuous eruption, the appearance of the teeth, and especially their apices, varies considerably throughout the life of a horse. Furthermore, due to variable eruption times, the length of the reserve crowns of the cheek teeth vary, with the first molars (09s) consistently being the shortest as the first permanent cheek teeth to erupt (36). Additionally, the second premolar teeth (06s) are shorter than the adjacent cheek teeth. The alveoli of the first and second maxillary premolar (06s and 07s) teeth do not extend into the paranasal sinus lumen and lie within the maxillary bone. However, the majority of the alveoli surrounding the remainder of the check teeth extend into the paranasal sinuses, with the exact relationship dependent on the tooth and age of the horse. The alveolus of the fourth maxillary premolar tooth (08s) is partially or fully within the rostral maxillary sinus (37). The alveoli of the first maxillary molar teeth (09s) frequently extend into the rostral maxillary sinus lumen (in horses over the age of 6 years all extended into the rostral maxillary sinus lumen). The second maxillary molar teeth (10s) either extend into the rostral or caudal maxillary sinus and the third maxillary molar teeth (11s) always extend into the caudal maxillary sinus (37). With increasing dental age and gradual decrease in length of the reserve crown, the alveoli of the caudal root of the maxillary 09s and the maxillary molars protrude less into the paranasal sinuses and thus move ventrally from the infra-orbital canal. Additionally, by 15 years of age, dental drift causes the apex of the Triadan 11s to move rostral to the orbit, resulting in increased distance from the infraorbital canal, smaller sized crowns and decreased width of the interdental space (37).

#### Apical or Tooth Root Infection

**Figure 4:**
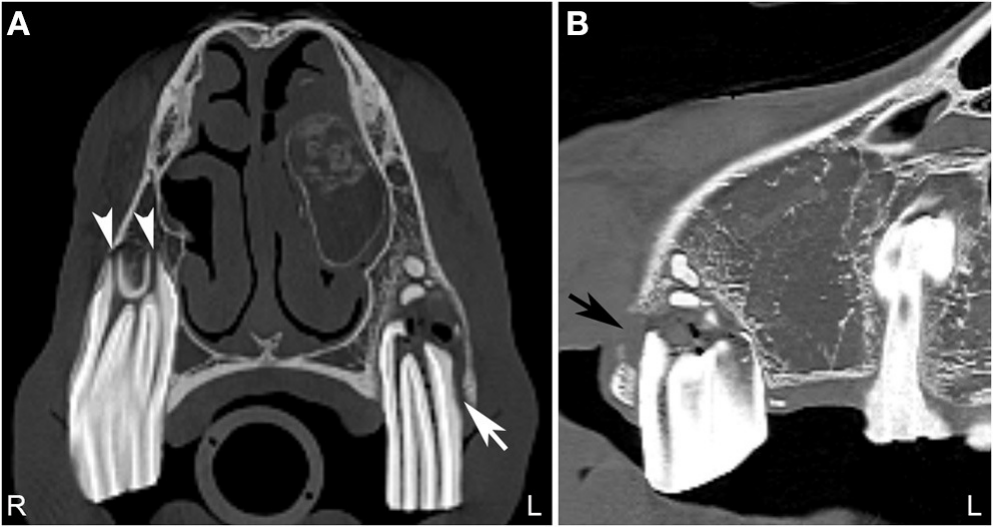
Normal tooth and apical abscess. Transverse **(A)** and sagittal **(B)** computed tomography image at the level of the second maxillary premolar teeth displayed in a bone window of a 7-year-old Quarter horse, which had a fractured left third maxillary premolar tooth (Triadan 207) removed at 4 years of age. She recently developed a swelling in the area of the left maxilla. On the right side, the periodontal space surrounding the second maxillary premolar tooth (106) is of even width and the apical foramina (white arrowheads) are small. Good definition of the infundibular enamel and peripheral cementum of the right maxillary premolar tooth is noted **(A)**. On the left side **(A,B)**, the apical foramina are increased in size and the periodontal space is widened (white arrow). The left second maxillary premolar tooth (Triadan 206) roots are blunted and gas is noted in the soft tissue attenuating tissue adjacent to the abnormal root. An area of lucency it noted surrounding the abnormal 206. These findings are consistent with an apical abscess. Small, well-defined mineral attenuating structures are noted at the distal aspect of the left abnormal maxillary premolar tooth, likely representing multiple small cemental depositions (“cement pearls”) secondary to chronic apical infection. At the rostral aspect of this tooth, a loss of the maxillary bone adjacent to the tooth root is noted (black arrow). An ovoid, soft tissue attenuating, small mass lesion surrounded by a thin-mineral attenuating rim with central amorphous mineral attenuating structures is noted in the left nasal cavity. This mixed attenuating lesion extends to the left maxilla at the site of the previous surgery for tooth removal and is suggestive of a chronic inflammatory process. This lesion displaced, but was not originating from the ventral nasal conchal bulla. The 207 is absent. L, left; R, right.

The tooth root in horses forms over time, and in young horses, where the tooth root is not developed, the term apex is preferred. The term apical infection can be used in horses of all ages. There are various causes of apical dental infection that are usually secondary to pulpar infection, trauma, and abnormally positioned teeth (38, 39). Common CT findings in horses with apical infection are similar to radiographic findings and include widening of the periodontal space, loss or irregularity of the lamina dura, sclerosis of the alveolar bone surrounding the tooth apex with or without osteolysis, blunting of the tooth roots, gas within the pulp or adjacent to the tooth root and fragmentation of the tooth roots (Figure 4). Loss of lamina dura without any other changes related to an apical infection should not be interpreted as a definitive sign of apical infection as the thinness of the lamina dura and/or lack of adequate image resolution may limit visualization of the lamina dura also in normal horses (40). Additionally, the presence or absence of the lamina dura did not affect the outcome of intraoral tooth extraction (41).

**Figure 5:**
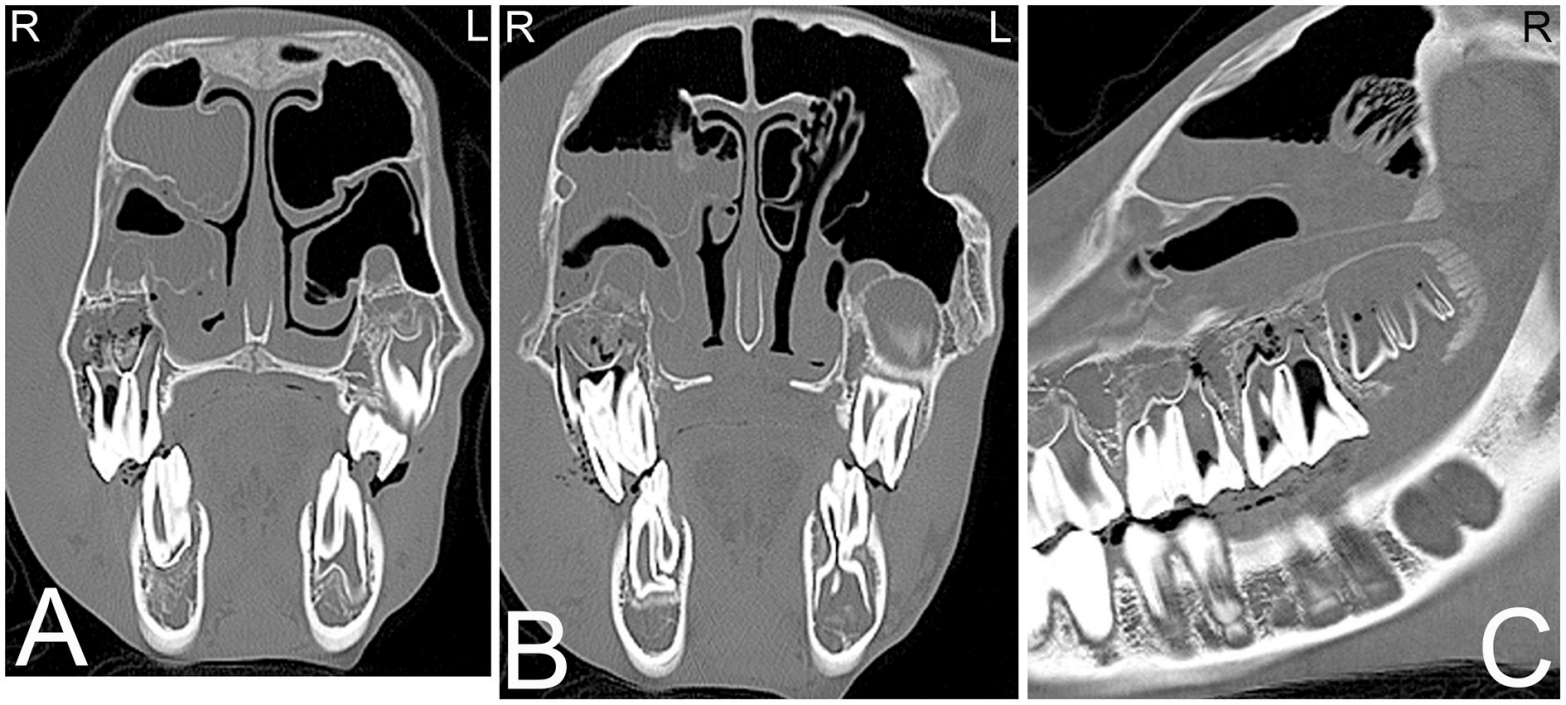
Apical abscessation of multiple deciduous erupted and unerupted maxillary cheek teeth. Transverse **(A,B)** and sagittal **(C)** computed tomography images displayed in a bone window of the rostral to mid aspect of the skull of a 2-month-old Fox Trotter foal with a history of swelling of the head for 4 days. Diffuse, small gas opacities are seen within and surrounding the apices of the right maxillary deciduous fourth premolar (Triadan 508), the deciduous third maxillary premolar, and the unerupted permanent first maxillary molar teeth. A moderate amount of dependent fluid is noted in the right maxillary and ventral conchal sinus consistent with sinusitis. Mild widening of the periodontal space around the apices of the mandibular cheek teeth with mild periosteal remodeling along the ventral aspect of both mandibles is present. Marked soft tissue swelling is noted along the entire right side of the head. Culture of sinus fluid and histopathology revealed a severe chronic osteomyelitis of the maxillary bone with fibrinous cellulitis and vasculopathy secondary to a mixed bacterial infection including *Staphylococcus* and *Clostridium*. L, left; R, right.

Infections of the rostral three maxillary cheek teeth result more frequently in maxillary swelling (Figure 3) and draining tracts than infections of the caudal three maxillary cheek teeth, which are more likely to result in nasal discharge (42). Since the caudal root/apex of the fourth maxillary premolar and the molar tooth roots are closely associated with the maxillary sinus, dental disease can result in sinusitis. Infection of the apical aspect of the mandibular teeth results most frequently in mandibular swelling and draining tracts, and tends to most frequently occur around the time of eruption of these teeth (42). Infection of more than one tooth are rare but can occur in horses of all ages (Figure 5).

**Figure 6:**
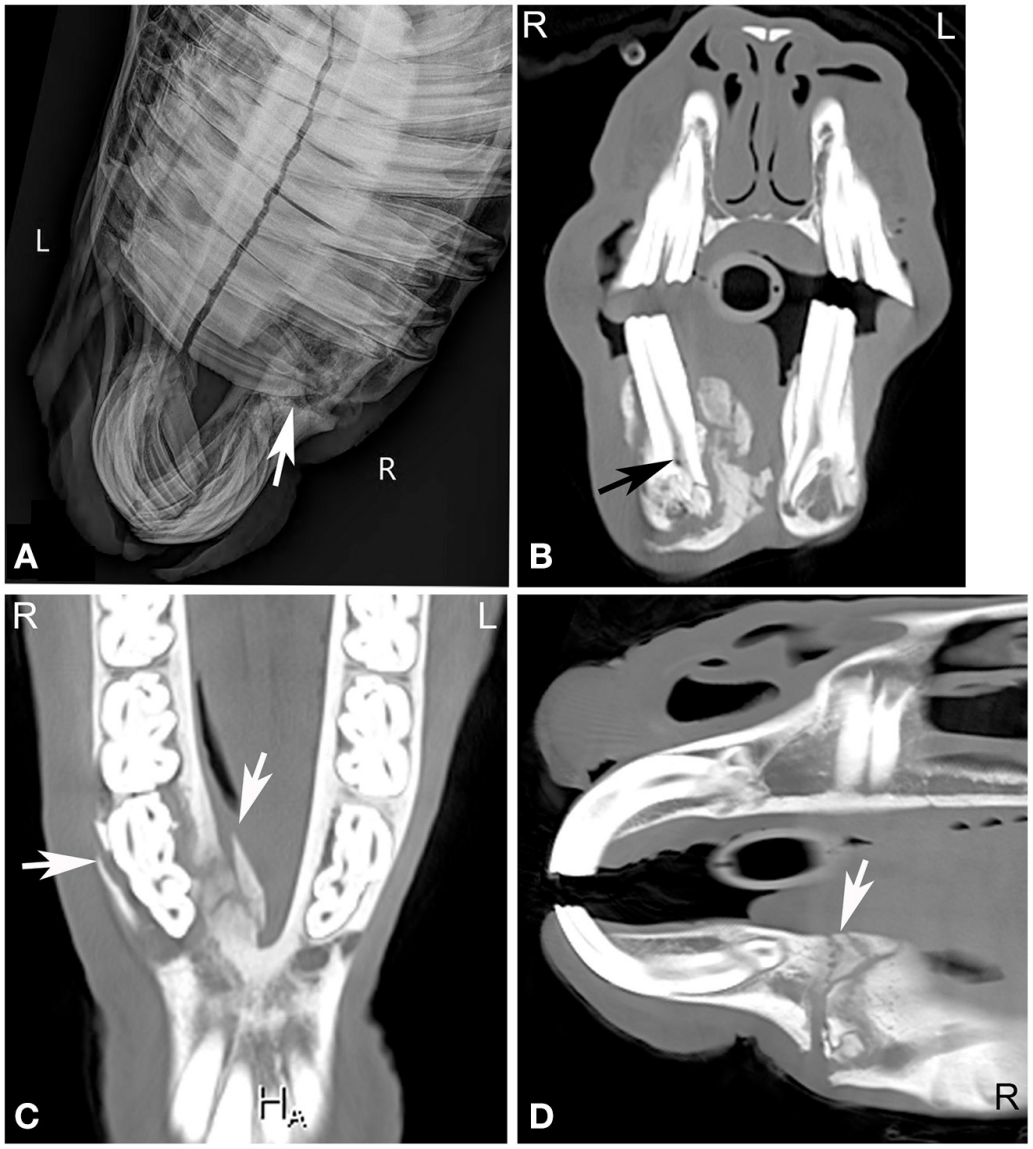
Chronic mandibular fracture with cheek tooth apical infection. Radiographic and CT images of a chronic fracture of the right mandible with involvement of the right mandibular premolar teeth in an 8-year-old Miniature horse. Right dorsal to left ventral oblique radiograph **(A)**, transverse **(B)**, dorsal **(C)** and sagittal **(D)** CT images of the rostral aspect of the head displayed in a bone window reveal a chronic right mandibular fracture (**A,C,D**, white arrows) with moderate osseous remodeling and involvement of the right second mandibular premolar tooth (Triadan 406). Gas is noted in the pulp of Triadan 406 (**B**, black arrow). *Actinomyces* species were cultured from tooth after its extraction. L, left; R, right.

In a study comparing the CT and radiographic findings in 32 horses with dental disease, CT had a reported sensitivity of 100%, specificity of 96.7% and a positive predictive value of 88.9% for detecting which tooth was affected. This was considerably higher than radiography (sensitivity: 72.5%; specificity: 89.5%; positive predictive value: 64.4%). CT detected a higher number of diseased teeth, and agreement for the specific tooth affected was only about 50% between the two methods (37, 43). In another study where dental sinusitis was present, dental disease was diagnosed in only 57% of horses on the initial radiographs (1). The diagnosis of sinus involvement in combination with dental abnormalities using CT is relatively uncomplicated and accurate, since there is no overlap of surrounding anatomic structures. Although rostral maxillary apical infections are usually easily diagnosed on radiographs (Figure 6), caudal cheek teeth apical infections can be challenging and therefore CT should be considered especially in cases of suspected dental sinusitis, when no response to treatment is noted and no definitive radiographic changes are seen (34, 43).

#### Dental Fractures

Dental fractures commonly occur secondary to non-traumatic causes such as infundibular caries, apical infection or “idiopathic” dental fractures (44–47). “Idiopathic” and infundibular caries-related fractures are more commonly seen in maxillary than mandibular teeth (46). Head trauma frequently involves the rostral mandible resulting in fractures of the rostral mandibular cheek teeth above or below the gingiva. Dental fractures can also result from iatrogenic trauma such as dental procedures, but this is less common. Idiopathic fractures of the cheek teeth have been described with a prevalence as high as 5.9% (48, 49). Characteristic patterns of idiopathic maxillary cheek teeth fractures include lateral “slab” fractures, which are reported in up to 45% of teeth with idiopathic fractures, followed by midline infundibular caries-related sagittal fracture and other patterns. In the mandibular cheek teeth, idiopathic fractures are less commonly reported and include lateral slab fractures, which comprise about 12% of idiopathic mandibular cheek teeth fractures, followed by various other patterns (45). Apical fractures can be challenging to diagnose radiographically, but are usually readily identified using CT. In one study, only approximately one third of tooth fractures were diagnosed using radiography compared to CT. Furthermore, CT also identified more teeth as abnormal than was clinically suspected (34).

#### Equine Odontoclastic Tooth Resorption and Hypercementosis

Equine odontoclastic tooth resorption and hypercementosis is well-documented progressive disease in older horses commonly affecting only the incisor and canine teeth. Despite this being a painful disease, it is often undiagnosed. The etiology of the disease is unknown (50, 51). EOTRH is characterized by either the predominately resorptive or hypercementosis pattern, or by a combination of both patterns. Typically, on radiographs, a bulbous enlargement of the intraalveolar crown root, resorption of the reserve crown, root and apex with widening of the peridontal ligament space as well as tooth fractures are noted (51). Teeth affected by EOTRH frequently have endodontic disease with inflammation, lysis, necrosis and fibrosis of the pulp (51).

#### Endodontic Disease

**Figure 7:**
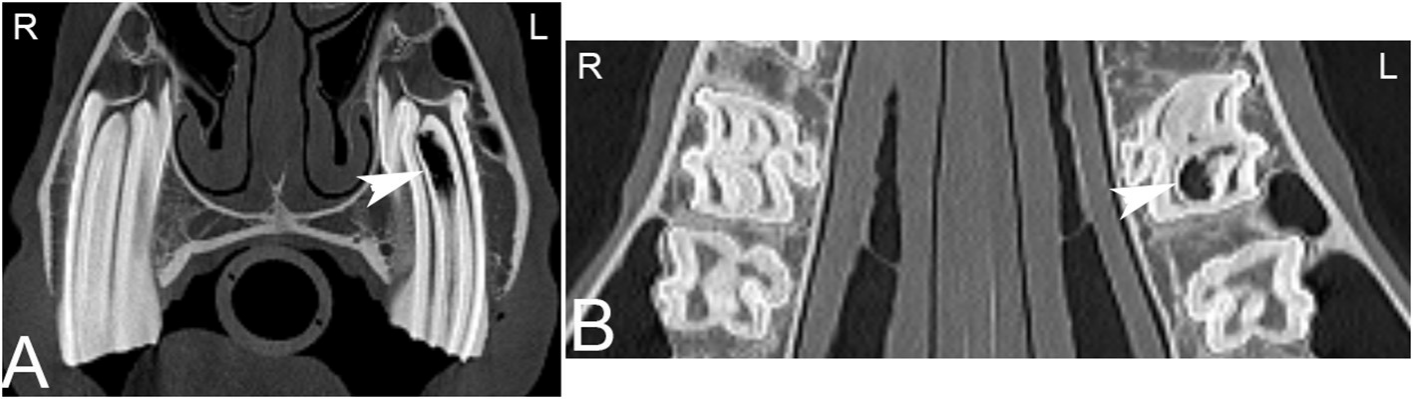
Apical infundibular cemental hypoplasia. Transverse **(A)** and dorsal **(B)** computed tomography images displayed in a bone window in a 14-year-old pony mare. Gas is present in the apical aspect of the infundibulum of Triadan 209 (white arrow heads). L, left; R, right.

CT is considered an excellent technique to evaluate individual teeth for the presence of endodontic disease i.e., pulpar disease (Figure 7), which can be missed on conventional radiographs (14, 52, 53). Endodontic or pulpar disease usually precedes apical disease and has been discussed under apical infection above. Scintigraphy is also useful in assessing pulpar/apical infections (54) and may provide additional clinically important information in early dental disease.

#### Dental (Odontogenic) Tumors

Dental tumors are rare; however, they are reported more frequently in equids than in other species. Odontogenic tumors tend to be locally invasive but seldom metastasize. Common odontogenic tumors include ameloblastomas, odontomas and cementomas; all these tumor types have multiple subcategories. Ameloblastomas tend to occur more commonly in the mandibular teeth of adult horses from 5 to 20 years old, but they have also been reported in foals (55). They tend to be locally destructive, round to multilobulated in shape and often expansile in their growth pattern (56). Odontomas occur more commonly in younger horses, and ameloblastic odontomas can occur in horses as young as 6-weeks-old. Odontomas frequently involve the maxilla causing regional swelling and displacement of the teeth (56). Cementomas are reported to involve the incisors and premolars; however, there has been overlap in the literature between actual neoplastic (true) cementomas and dysplastic conditions related to the cementum (e.g., hypercementosis) (57). Radiographs often identify the presence of odontogenic tumors, but the extent of these lesions is frequently better evaluated using CT, that provides additional information for surgical planning or radiation treatment (19, 58). CT features of odontogenic tumors include maxillary or mandibular expansion, alveolar and cortical bone lysis and variable degrees of mineral attenuating content. In one report, complex odontomas were differentiated from other odontogenic tumor types due to the presence of enamel attenuation within the mass (55).

#### Oligodontia, Polydontia or Dysplastic Teeth

Reduced number of or presence of additional teeth are often first noted on clinical examination. Hypodontia, oligodontia and anodontia, which describe the lack of one tooth to multiple teeth, can cause abnormal occlusion and therefore abnormal ware of other teeth. In a study of 3 horses with hypodontia, the maxillary fourth premolar was missing in all three horses (59). In polydontia, which is the presence of extra or supernumerary teeth, the teeth may have a normal anatomical shape or can be malformed. When teeth are dysplastic, which is not uncommon in horses, the teeth can be so abnormally shaped that it can be difficult to decide if an apical infection is present or not. In these cases, CT examination may enhance differentiation between abnormally shaped teeth with or without apical inflammation.

### Other Dental Imaging Findings in the Equine Head

#### Dentigerous Cyst (Ectopic Tooth, Ear Tooth, Temporal Teratoma, Heterotopic Polydontia)

**Figure 8:**
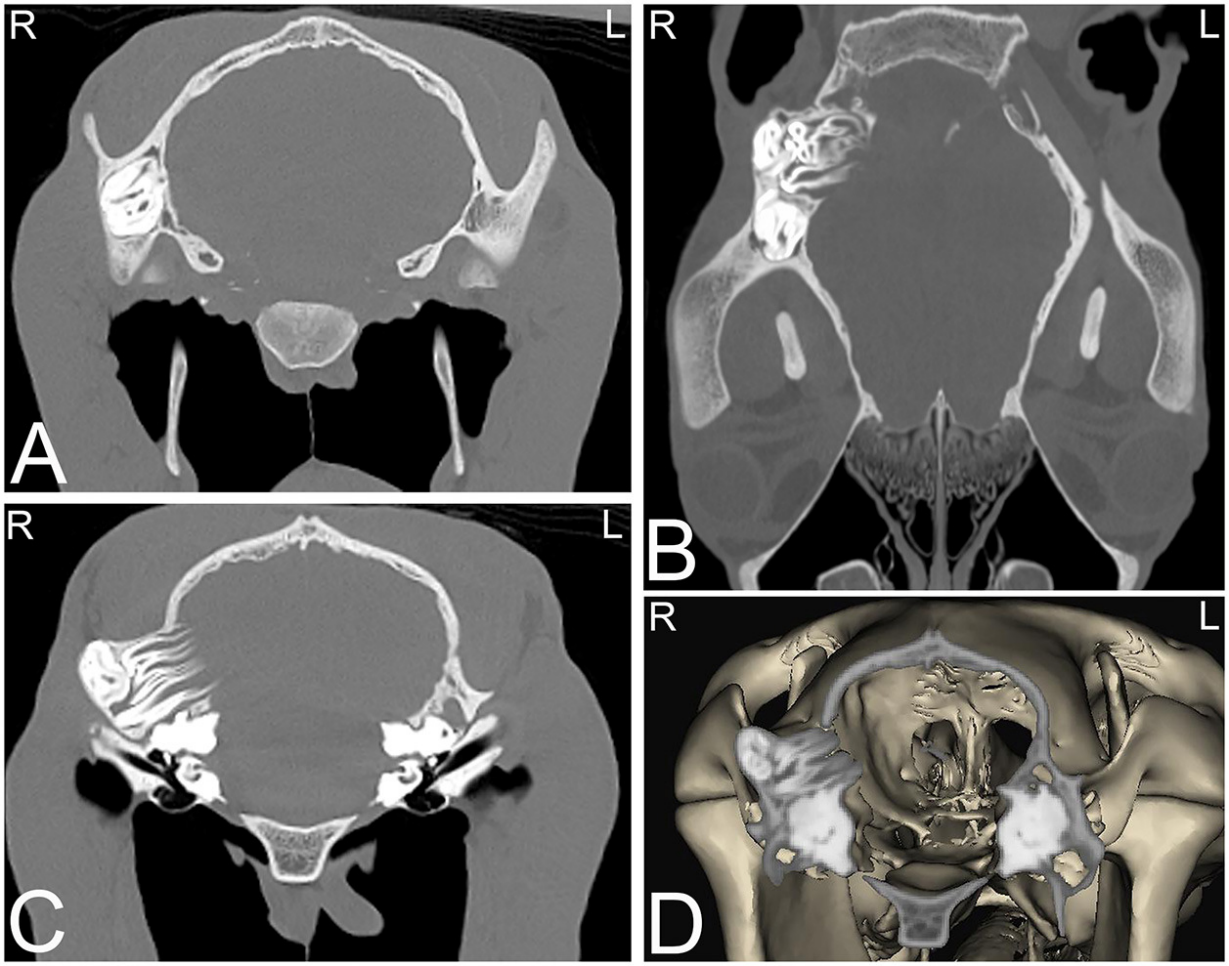
Dentigerous cysts. Computed tomography images of the caudal aspect of the head of a 2-year-old Morgan gelding with a history of a draining tract of 4–5 months duration by the right ear. Transverse **(A,C)**, dorsal **(B)**, and 3-dimensional **(D)** CT images reveal three abnormally positioned and abnormally shaped tooth-like structures at the base of the right ear with remodeling of the right temporal and occipital bones consistent with dentigerous cysts. Two of these tooth-like structures have their roots extending toward the calvarium but no neurologic abnormalities were reported. L, left; R, right.

Typically, this is a benign developmental abnormality resulting from incomplete closure of the first branchial cleft, resulting in the development of a cystic structure surrounding a tooth, often close to or even attached to the temporal bone. Frequently, these cystic structures are associated with a draining tract by the pinna of the ear and can pose a cosmetic problem (60). They can be present unilaterally or bilaterally. Radiographs often detect the abnormally positioned tooth-like structure; however, the associated temporal bone involvement is often not evident on radiographs, and cross-sectional imaging such as CT or MRI is required to completely evaluate the extent of the lesion (Figure 8).

### Sinonasal Disease

The equine paranasal sinuses are a complex system that consists of paired rostral and caudal maxillary, frontal, ventral and dorsal conchal, ethmoid and sphenopalatine sinuses. The frontal and dorsal conchal sinus are often grouped together and called the conchofrontal sinus. The conchofrontal, caudal maxillary, ethmoidal and sphenopalatine sinuses communicate with each other and the rostral maxillary and the ventral conchal sinuses interconnect with each other (61). The paranasal sinuses are incompletely visualized using radiography because there is a large amount of superimposition. The sphenopalatine sinus with its variable anatomy is incompletely visualized using radiography, and often requires CT for more complete assessment. Overall CT allows complete assessment of the paranasal sinuses, which contributes to diagnostic, therapeutic and prognostic information (62).

The most common sinonasal diseases in horses include primary sinusitis, dental sinusitis secondary to apical infection, sinus cysts, ethmoid hematomas and neoplasia. Intra-sinus masses are more frequently sinus cysts, ethmoid hematoma and inflammatory nasal polyps than neoplasia. Neoplasia of the sinus system in horses is quite rare, and tumors originating from the oral cavity, osseous or dental structures extending into the sinuses are more common than tumors originating within the sinuses (15, 63).

#### Sinusitis

**Figure 9:**
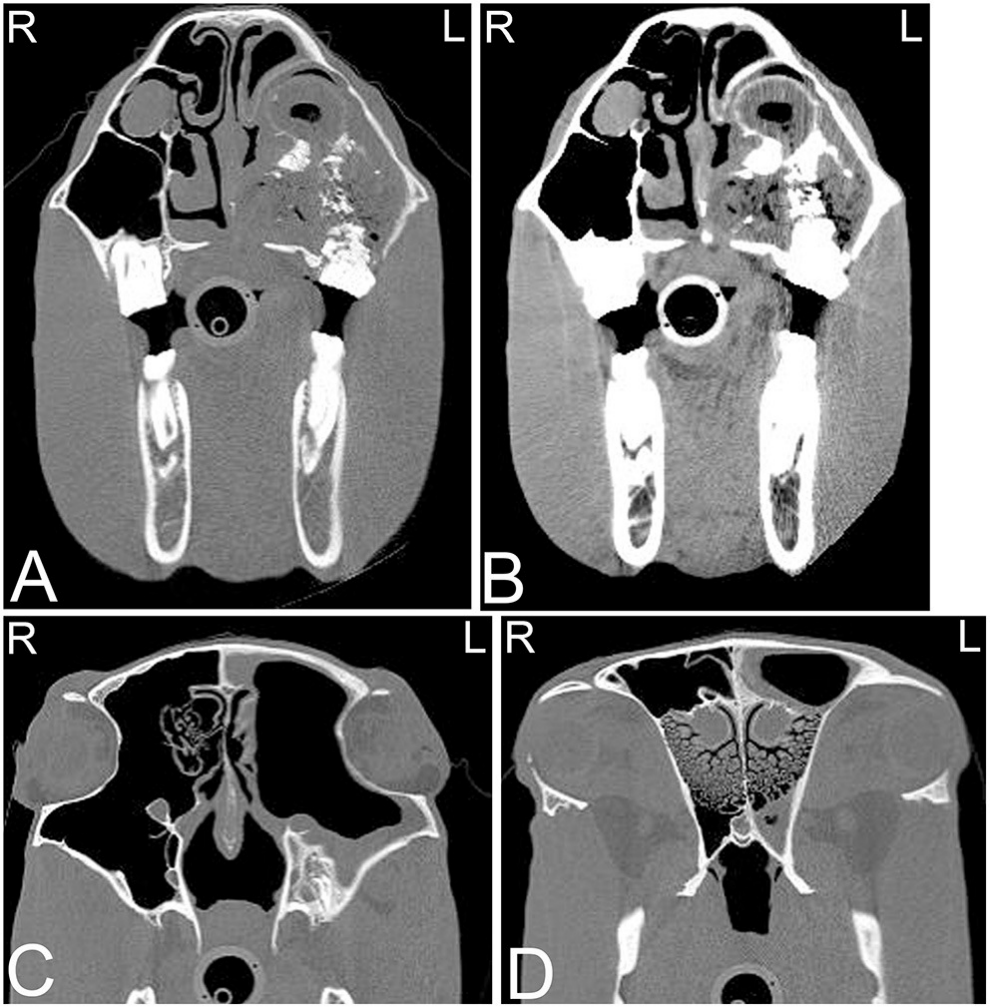
Chronic lymphoplasmacytic sinusitis and right oronasal and oromaxillary sinus fistula. Transverse computed tomography images of the rostral **(A,B)** and caudal **(C,D)** aspect of the skull of a 20-year-old Paint horse with a large diastema between the right second and third maxillary molar teeth (Triadan 110 and 111), and a 2-year history of nasal discharge, which improved but not resolved with antibiotic treatment. In the rostral CT images in a bone **(A)** and soft tissue window **(B)** a heterogeneous soft-tissue attenuating mass with diffuse gas is noted surrounding the apices of Triadan 110 and 111 that is extending from the occlusal surface (white arrow) into the right maxillary sinus filling the entire rostral and part of the caudal right maxillary sinus. The roots of these two teeth are blunted and irregular, and the surrounded alveolar bone is disrupted. The right nasal passages are occluded by the mass. In the CT images of the caudal aspect of the skull displayed in a bone window **(C,D)**, a marked thickening of the mucosal lining of the left nasal cavity and left maxillary sinus is noted. Osteitis of the left maxillary bone with disruption and remodeling of the infraorbital canal is present. On histopathology a chronic lymphoplasmacytic sinusitis and rhinitis was noted. L, left; R, right.

In primary or dental sinusitis, an increased amount of soft tissue attenuating material and reduced amount of air filling is noted in the sinus, associated with exudation caused by bacterial infection (Figure 9). Assessment of the cause and location of the sinus inflammation is important to assess to establish treatment plans and prognosis and frequently requires imaging. Horizontal fluid lines within the sinus may be noted on radiographs as well as on CT images, with the orientation of these lines dependent on patient position (standing vs. recumbent). The most commonly affected sinuses involved are the rostral maxillary and ventral conchal sinuses, which are affected in over 90 and 87% of horses with sinus disease. The caudal maxillary and dorsal conchal sinuses are affected in more than 50%, and the ethmoid and sphenopalatine sinuses were less commonly affected in horses with sinus disease (64). Additionally, osseous remodeling and enlargement of the osseous paranasal structures commonly occurs secondary to inflammation of the paranasal sinuses (65, 66). Considering the complex nature of the paranasal sinuses, and their intimate anatomical relationship with nasal structures, secondary nasal disease is commonly reported in horses with sinus disease. In addition to the remodeling and fluid filling of the paranasal sinuses, horses with nasal involvement have nasal mucosal swelling, sino-nasal fistulas in addition to empyema and remodeling of the nasal conchal bullae (64). Commonly, when disease of the nasal conchal bulla is present, sinus disease is present; however, empyema without concurrent sinus disease has been reported in rare instances (67). Furthermore, mineralization of the infraorbital canal and changes in size and shape of the canal were reported in 78% of horses with sinus disease (68). Dental disease is the most commonly reported cause of sinusitis and was noted in more 50% of the horses with sinusitis at three centers (64). Sinus mycosis can also occur in horses, and causative fungal agents include *Cryptococcus neoformans, Blastomyces dermatides, Aspergillus* spp., and others. Sinus mycosis can be associated with fluid accumulation, turbinate and conchal destruction, and remodeling of the surrounding bones. However, in some cases, sinus mycosis may only cause minimal increase in soft tissue attenuating material in the nasal cavity and paranasal sinuses and can therefore easily be missed on radiographs. Usually, sinus mycosis has a classical appearance using sinoscopy; however, if osseous involvement is suspected, CT provides a better overview of the extent of the lesion. On CT, small soft tissue attenuation plaques may be noted in the nasal cavity and paranasal sinuses, in addition to mucosal thickening, fluid within the nasal cavity and sinuses and remodeling of osseous structures.

Radiographs should always be carefully evaluated in horses with sinus disease, to see if more than one sinus is involved. On radiographic studies, sinus involvement is more likely to be diagnosed when involvement of the maxillary or frontal sinuses is present; sphenopalatine or ventral conchal sinus involvement are challenging to diagnose (34). Radiography and CT have demonstrated good agreement for diagnosing sinusitis when the mucosa of the sinuses measures more than 1 cm in thickness and fluid is present within the sinus or when a complete sinus obstruction is present. In cases where no fluid is present in the sinus and the mucosa is not greatly thickened, diagnosis by radiography is challenging. Furthermore, though radiography is a reliable technique for diagnosing sinusitis when fluid is present, it is often inadequate in diagnosing the cause of sinusitis and the involvement of the various compartments. In horses, secondary sinusitis due to dental disease is common, and on radiographs the visualization of the reserve crown and apex of the teeth may be hindered by the presence of fluid in the sinuses, mucosal thickening and osteitis of the adjacent bone. CT is extremely beneficial in these cases.

#### Sinus Cysts (Paranasal Sinus Cysts)

**Figure 10:**
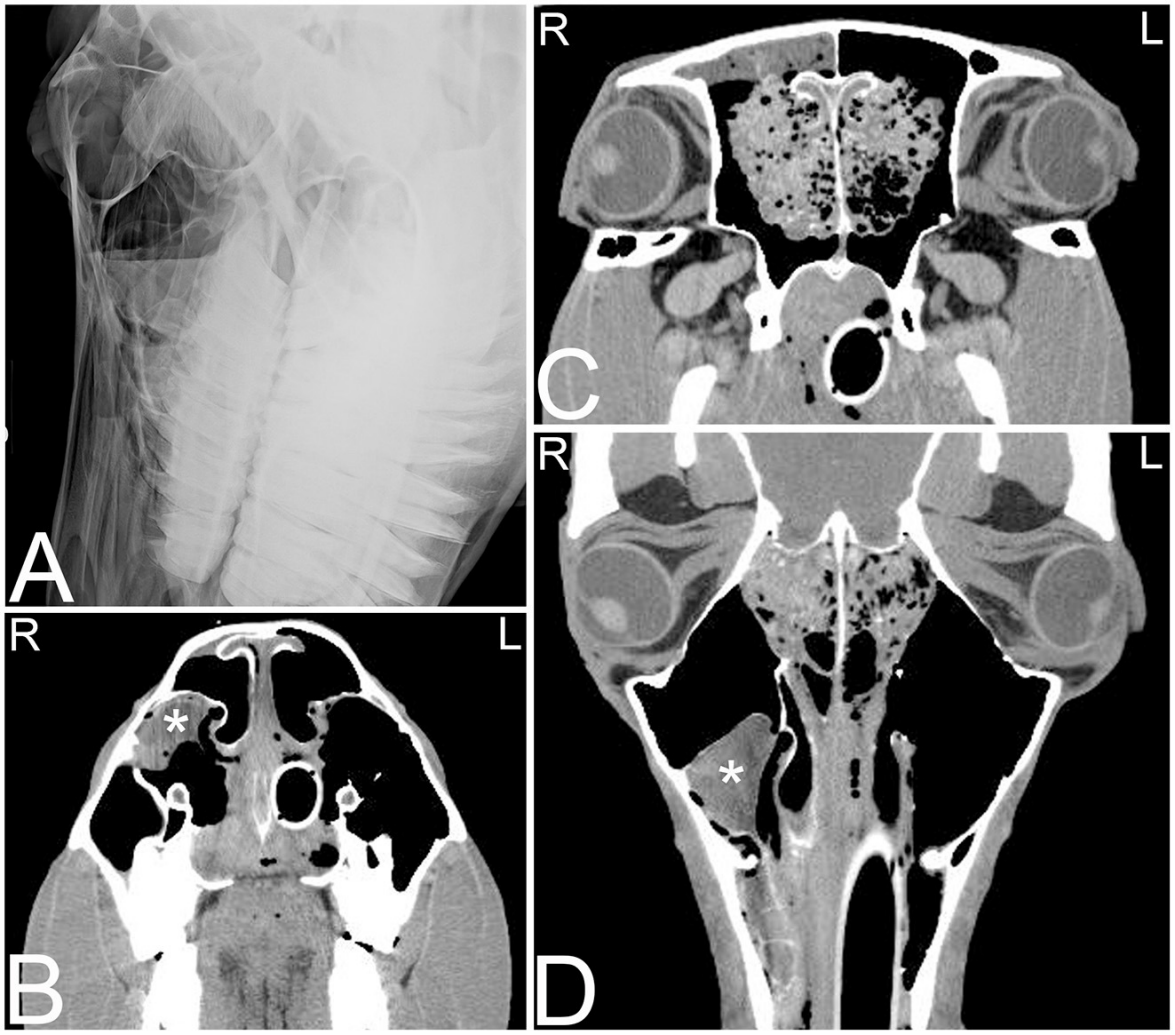
Sinus cyst and ethmoid hemorrhage. Radiographic and computed tomography images of the caudal aspect of the head of an 11-year-old Quarter horse with a history of ~1 year of unilateral mucoid nasal discharge. In the oblique radiographic view highlighting the right maxillary sinus area **(A)**, horizontal fluid levels are noted in the rostral and caudal right maxillary sinuses. The rostral aspect of the ethmoid turbinates is incompletely identified. In the transverse CT images at the level of the rostral nasal cavity **(B)** a thin walled, indistinct, heterogeneously contrast enhancing structure (marked by white asterisk) is noted in the dorsolateral aspect of the right maxillary sinus. In the transverse **(C)** and dorsal **(D)** CT images a heterogeneous contrast enhancement of the right ethmoid area with an increase in soft tissue attenuating material is noted. Additionally, a small amount of fluid attenuating material is noted in the right rostral and caudal maxillary and frontal sinuses. Histopathology of tissue obtained by biopsy during a sinoscopy from the right rostral conchal lesion suggested a sinus cyst with chronic sinusitis. The bilateral lesion of the ethmoid area was thought to be secondary to hemorrhage caused during intubation for anesthesia during the CT study. L, left; R, right.

**Figure 11:**
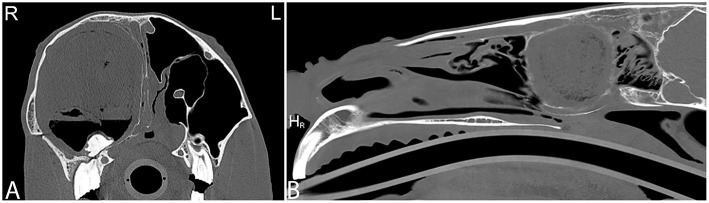
Large sinus cyst with maxillary distortion. Transverse and sagittal computed tomography images of the head of a 20-year-old Thoroughbred gelding with a history of facial swelling. In the transverse **(A)** and sagittal **(B)** bone window CT images of the mid-aspect of the skull, a large mixed attenuation, round, well-defined structure surrounded by a thin rim of mineral attenuating material with deformity of the right maxilla and ethmoid bone is noted. L, left; R, right.

**Figure 12:**
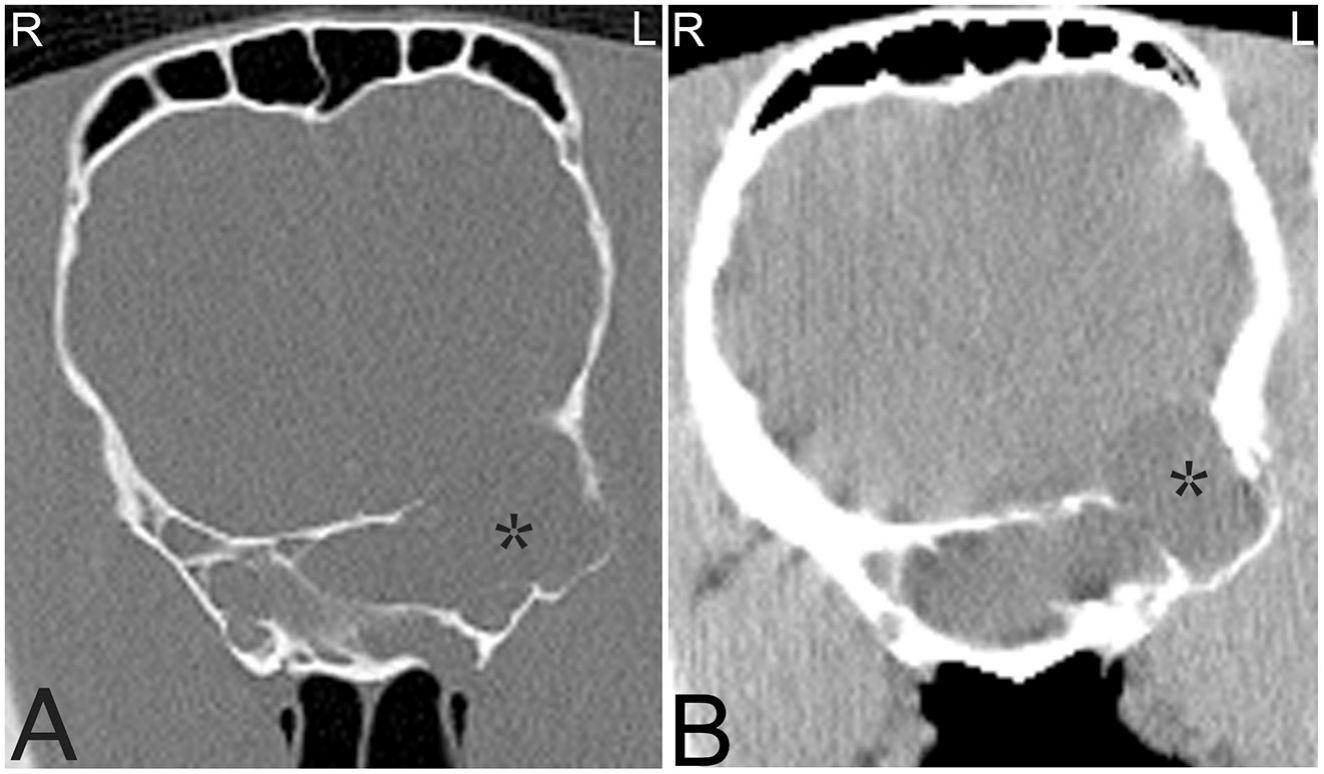
Large sinus cyst involving the sphenopalatine sinus. Computed tomography images of the head of a 5-year-old Thoroughbred mare with an acute onset of blindness of 3 weeks duration. Transverse bone window **(A)** and soft tissue window **(B)** CT images of the head at the level of the caudal aspect of the sphenopalatine sinus, illustrate a poorly defined, lobulated, destructive mass in the left sphenopalatine sinus with extension toward the neurocranium (stars). Contrast medium enhancement is not evident centrally but mild enhancement is present peripherally **(B)**. Histopathology revealed a sinus cyst with extension into the neurocranium thereby compressing the optic nerves at the chiasma. No signs of a neoplasia were noted on histopathology. L, left; R, right.

Currently, there is no known definitive etiology and pathogenesis reported for these progressively expansile paranasal sinus cysts, which do not spontaneously regress (69). Sinus cysts are radiographically often noted as a rounded, soft tissue attenuating mass within the nasal cavity or paranasal sinuses. Cysts may block drainage and also cause empyema of the affected sinus(es) with fluid lines. Frequently a thin mineralized capsule-like structure may be noted (Figure 10). If large cysts are present, distortion of adjacent frontal and maxillary bones may be seen in addition to deviation of the nasal septum (Figure 11). CT findings are similar to those reported radiographically; however, they allow better evaluation of the extent of the lesion with respect to adjacent bones, the nasal septum and paranasal sinuses (Figure 12). A recent study documented the presence of a hyperattenuating rim on pre-contrast CT images of paranasal sinus cysts. This is finding is not pathognomonic, but was present in the majority of sinus cysts. The average Hounsfield units of the cystic contents did not significantly differ from some other sinus diseases (ethmoid hematomas excluded); however, the majority of cystic contents were uniform in attenuation, which was not the cases in the other disease processes reported (17).

#### Progressive Ethmoid Hematomas (Hematomata)

**Figure 13:**
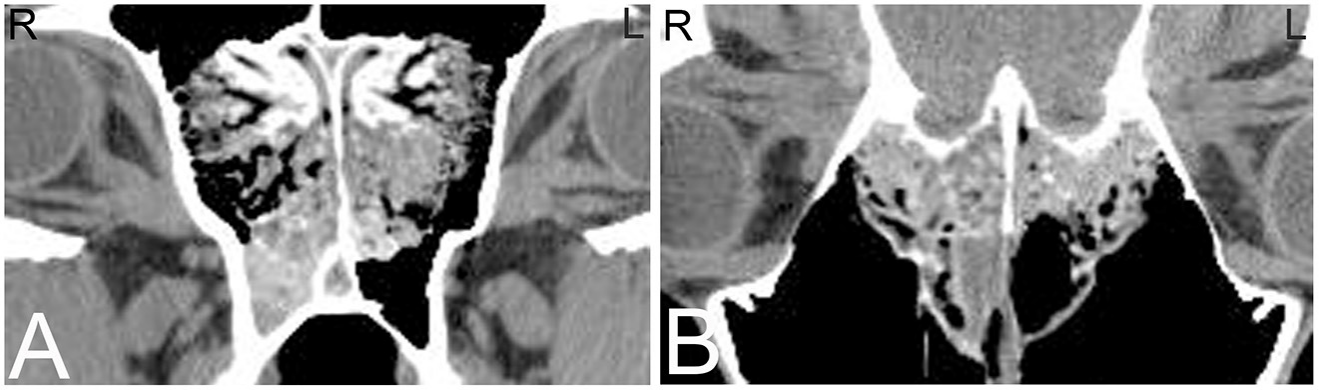
Progressive ethmoid hematoma. Computed tomography images of the head of a 10-year-old Warmblood gelding with a history of right-sided nasal hemorrhagic and purulent discharge. Transverse **(A)** and dorsal **(B)** CT images at the level of the cribriform plate reveal a large, minimally contrast enhancing mass at the right and to a lesser extent left ethmoid turbinates with extension into the spheno-palatine sinus, and multifocal regions of destruction of the cribriform plate; which is most consistent with an ethmoid hematoma or neoplasia. Histopathology confirmed an invasive ethmoid hematoma. L, left; R, right.

Intra-nasal progressive ethmoid hematomas (hematomata) (PEH) are readily diagnosed on nasal endoscopy, but intra-sinus hematomas require imaging or sinoscopy for diagnosis. Intra-sinus PEHs often appear radiographically as a well-defined, round to ovoid, soft tissue attenuating mass in the area of the ethmoid labyrinth, sphenopalatine or caudal maxillary sinuses. They arise in the area of the ethmoid labyrinth and progressively grow into the paranasal sinuses and nasal passages, following the path of least resistance. Ethmoid hematomas are more commonly seen unilaterally, but bilateral lesions have been reported (70). Although these can be diagnosed radiographically, when the lesion is small or when fluid is present in the sinus system, ethmoid hematomas may not be identified on radiographs or *via* sinus endoscopy (sinoscopy) (71). CT allows differentiation between fluid and a mass lesion and facilitates diagnosis of lesions of the ethmoid or sphenopalatine and maxillary sinuses, even if they are small. CT contributes to treatment planning because if the complete extent of paranasal sinus involvement is not diagnosed prior to treatment, surgical removal may be incomplete (71). Although radiographic and CT findings of ethmoid hematomas are non-specific and can appear similar to neoplasia (Figure 13), a mass with a mixed, hyperattenuating, swirling pattern without severe destruction or deformation of the adjacent bone suggests an ethmoid hematoma is highly likely (71).

#### Sinonasal Neoplasia

Sinonasal neoplasia is rare in horses, however, several tumors of the nasal cavity and/or paranasal sinuses have been described, with the most common being squamous cell carcinoma. Other reported tumor types in this location include neuroendocrine tumors, carcinoma, myxosarcoma, adenocarcinoma, and hemangiosarcoma (56, 63, 72). Most of these tumors are aggressive in nature and can lead to moderate to marked lysis of adjacent osseous structures including the cribriform plate. Occasionally inflammatory nasal polyps occur and do not usually show aggressive behavior. It had been suggested that some of these tumors are more likely to occur in certain anatomical sites, with most equine paranasal sinus tumors arising from the maxillary sinus in one study (72); however this was not demonstrated in a later study where the conchofrontal sinus was most commonly involved (19). Certain tumor types may display a preference for specific anatomic locations. Squamous cell carcinoma and unspecified carcinomas often arise in the maxillary sinus, whereas adenocarcinoma tends to develop in the nasal cavity and in the area of the ethmoid bone (73). Additionally, involvement of multiple sinuses is possible and therefore determining the origin of primary paranasal sinus neoplasia is challenging even with CT (19). Radiographs are usually inadequate to identify masses involving the sphenopalatine sinus and for demonstrating extension into the cranium; CT can overcome these limitations and provide more accurate information in regards to the extent, location and features of malignancy. On CT, these lesions are usually contrast enhancing, providing the potential to differentiate tumor borders from adjacent soft tissue structures and sinonasal fluid, allowing better treatment planning and prognostication. Furthermore, tumors may cause destruction of the adjacent osseous structures more commonly, compared to sinusitis, where osseous destruction is rarely noted. Additionally, CT provides the opportunity to measure the attenuation of a structure, therefore allowing differentiation between soft tissue masses and fluid (19).

### Osseous and Joint Disease of the Equine Head

#### Head Trauma

**Figure 14:**
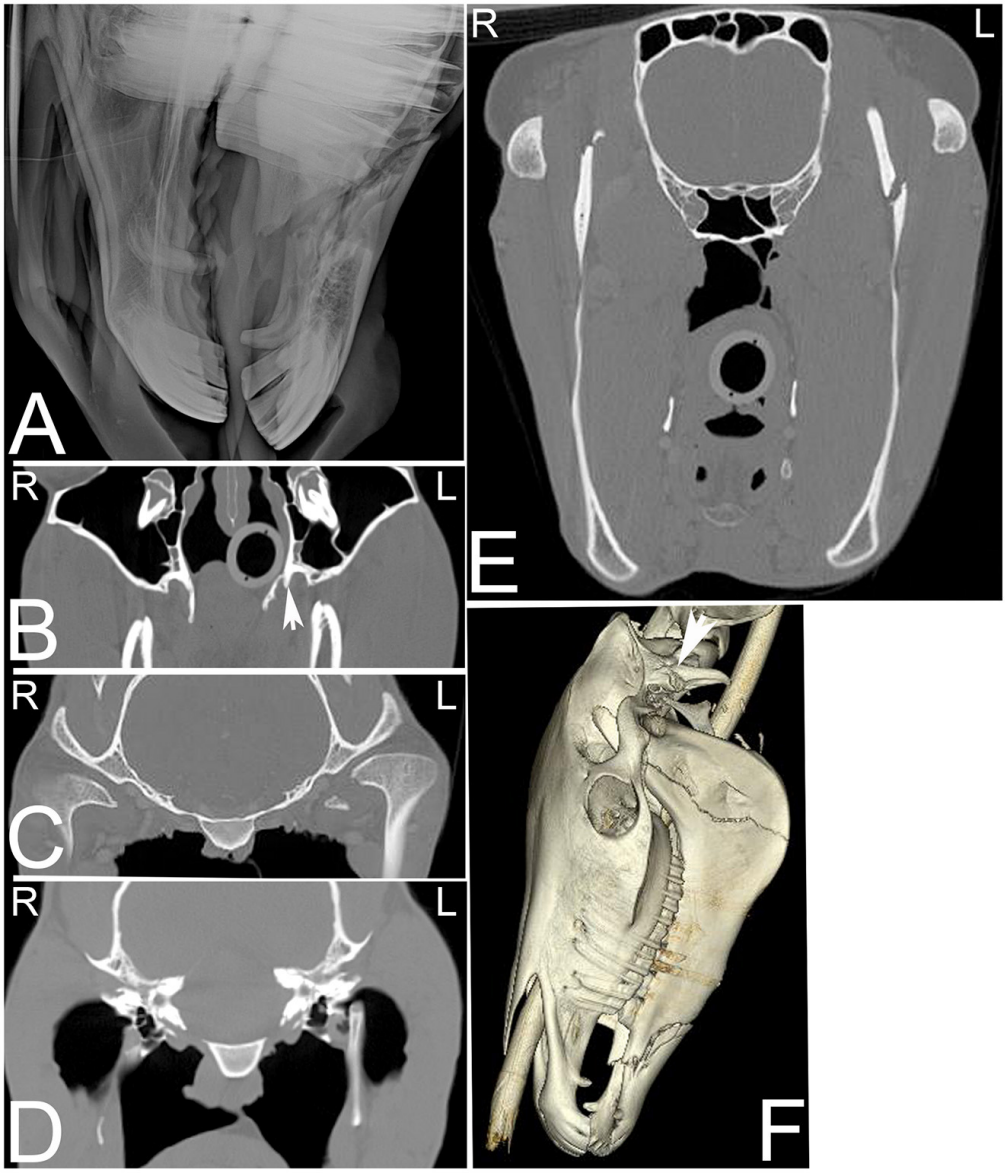
Extensive head trauma. Radiographic and computed tomography images of the head of a 6-year-old American Saddle horse gelding with a history of acute trauma and swelling of the left side of the head and right eye region on physical examination. On the radiograph **(A)** a long oblique fracture of the body of both mandibles with involvement of the roots of both second mandibular premolar teeth is noted. On transverse CT images **(B–E)** displayed in a bone window additional fractures involving the left pterygoid process (**B**, arrow), medial aspect of the left condyloid process of the mandible **(C)**, the dorsal aspect of the left stylohyoid bone **(D)** and the ramus of the left mandible **(E)** are noted. On the 3-dimensional reconstructed CT image **(F)**, the fracture of horizontal ramus (body) of both mandibles and the vertical ramus of the left mandible as well as a fracture of the left paracondylar process (arrow) can be noted. L, left; R, right.

**Figure 15:**
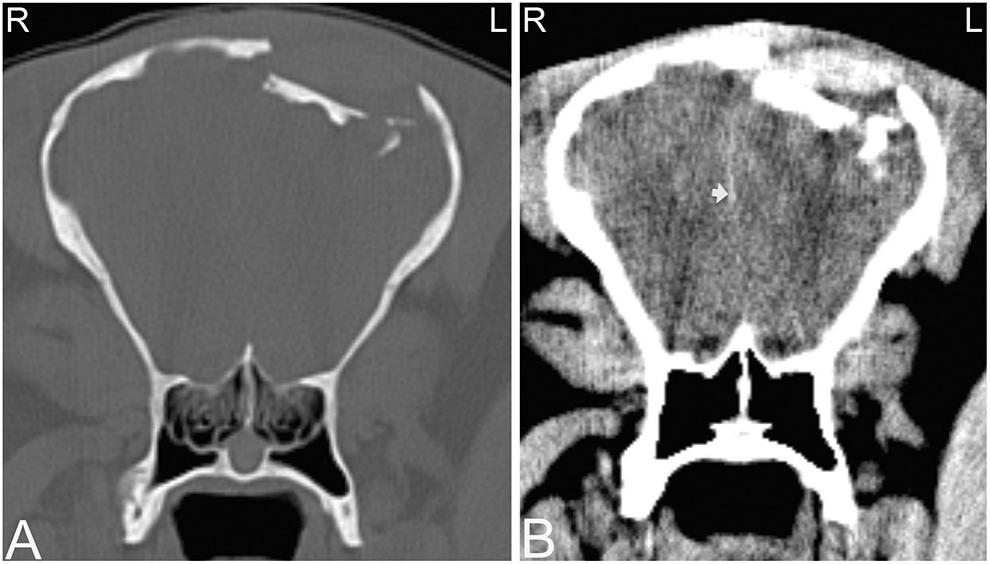
Compression skull fracture with brain hemorrhage. Computed tomography images of the head of a Miniature horse with a history of running into a wall and falling backwards. Transverse CT images displayed in a bone window **(A)** demonstrate a large, comminuted depression fracture of the left parietal bone with ventral displacement of several bone fragments into the brain. CT images displayed in a soft tissue window **(B)** illustrate large areas of patchy hyperattenuation in the left brain, which causes a mild rightwards displacement of the falx cerebri (white arrow). Similar areas of hyperattenuation are also seen in the soft tissues dorsal to the compression fracture. These hyperattenuating areas are most consistent with acute intracranial brain hemorrhage, and hematoma formation as well as hemorrhage and/or hematoma formation within the adjacent soft tissues. L, left; R, right.

Head trauma occurs frequently, especially in young horses and can result in asymmetry to the skull and nasal bleeding. There is good agreement between radiographic and CT findings in diagnosing fractures of external osseous structures of the skull; however, if the fracture extends toward the retrobulbar space and calvarium or when fractures are very comminuted, CT allows identification of more fractures and fracture fragments and provides more detailed information about fracture location (Figure 14). Additionally, 3-dimensional reconstructions may help assessing the full extent of the fractures (Figure 14F), which may be helpful especially when surgical intervention is considered. Particularly challenging locations to evaluate skull trauma radiographically include the temporomandibular joint, orbit and base of the skull (2). Basilar skull fractures occur in horses that fall over backwards (74), and the displacement of the fracture can be minimal, making radiographic diagnosis difficult. Although hemorrhage in the region of the guttural pouches may be detected radiographically, CT has proven useful for identification of the basioccipital-basisphenoid bone fracture and detection of fragmentation (75). Additionally, if brain trauma is suspected CT examination might reveal hypoattenuating areas of brain parenchyma suggestive of edema and areas of hyperattenuation relative to normal brain parenchyma suggestive of hemorrhage (Figure 15). Subdural or epidural hemorrhage can present as localized areas of hyperattenuation adjacent to the brain parenchyma often with displacement of the brain parenchyma (76). CT is an ideal modality for acute head trauma patients because it allows evaluation for fractures in combination with assessment of the brain for signs of hemorrhage. However, other disease processes of the brain are difficult to evaluate using CT and are better evaluated using MRI.

#### Nasofrontal Suturitis (Suture Separation, Periostitis or Exostosis)

**Figure 16:**
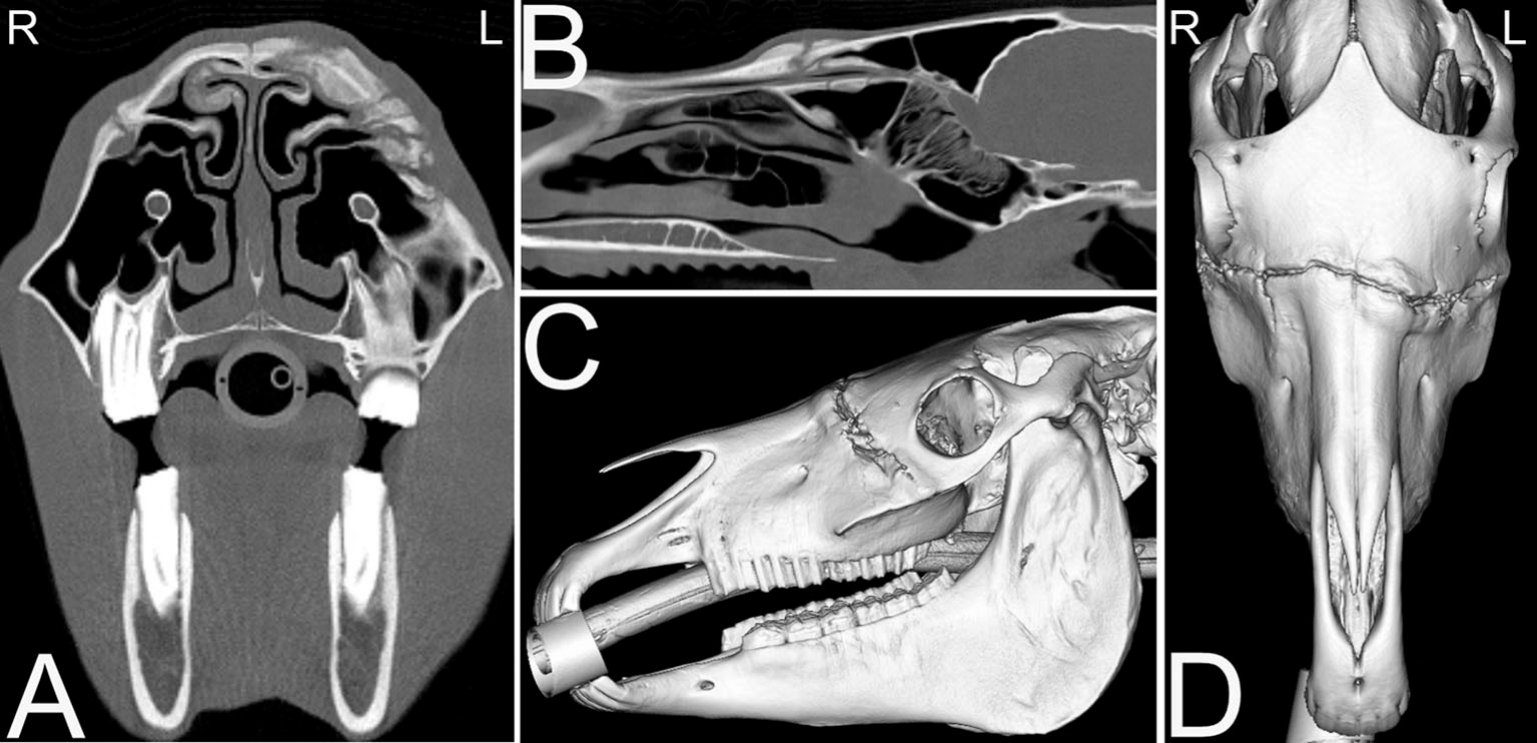
Nasofrontal suture exostosis. Computed tomography images of the head of a 14-year-old Quarter horse mare presented for facial swelling of ~1.5 months duration with possible history of facial trauma off a metal bar. On the transverse **(A)** and sagittal **(B)** CT images excessive osseous remodeling of skull at the level of the nasofrontal suture lines and other sites is noted. The remodeling is not exactly bilaterally symmetrical, and a traumatic cause is suspected. This remodeling is similarly noted on the sagittal **(C)** and dorsal **(D)** 3D CT images of the skull. Biopsy of the osseous remodeling of the suture revealed a pyogranulomatous periostitis. L, left; R, right.

Nasofrontal suture inflammation occurs most commonly in young horses, and usually affects the suture at the intersection of the frontal, nasal and maxillary bones. Suturitis of other facial bones especially the nasolacrimal and maxillolacrimal sutures also occur. Nasofrontal suture periostitis is frequently associated with swelling along the dorsal aspect of the skull and moderate cortical thickening, sclerosis and periosteal new bone formation along the frontal, nasal and maxillary bones adjacent to the suture. CT can be used to further evaluate the lesion, especially when involvement of the nasolacrimal duct is suspected, based on clinical signs (77–80). The etiology of nasofrontal suture separation is poorly understood, usually only of cosmetic concern and often self-limiting. It is suspected to be secondary to trauma (Figure 16) resulting in instability at the site of the suture line (81).

#### Osseous Tumors

**Figure 17:**
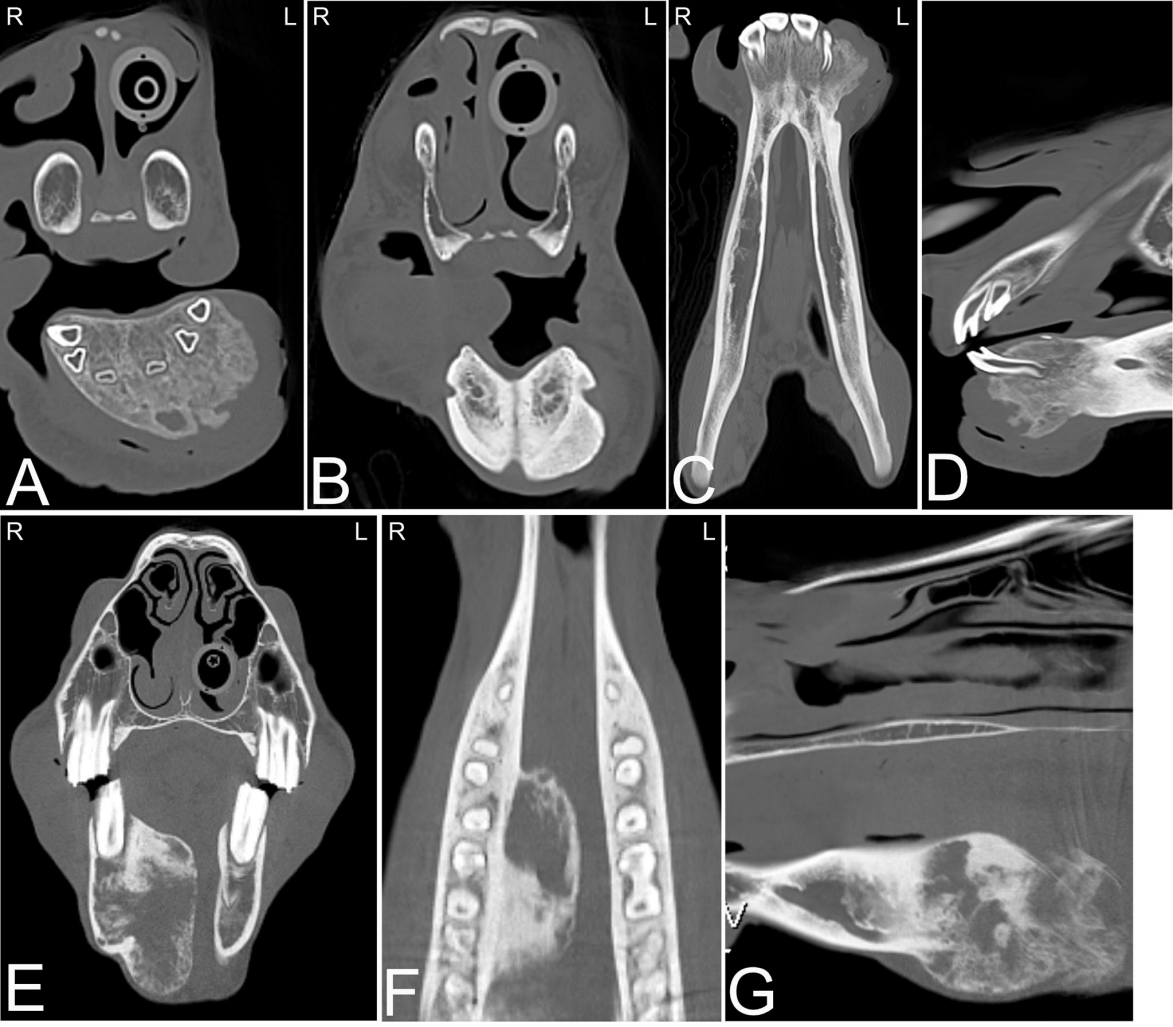
Ossifying fibroma and squamous cell carcinoma. Several head tumors can have a similar CT appearance. **(A–D)** Computed tomography images of the head of a 6-month-old horse presented for swelling around the rostral aspect of the mandible. Transverse **(A,B)**, dorsal **(C)** and sagittal **(D)** CT images displayed in a bone window reveal an area of indistinct osteolysis and osteoproliferative changes involving the most rostral aspect of both mandibles. No dental abnormalities were noted. The rostral aspect of the mandible was removed surgically and histopathology confirmed an ossifying fibroma. **(E–G)** Computed tomography images of the head of a 22-year old American Paint horse presented for swelling in the mandibular area. Transverse **(E)**, dorsal **(D)**, and sagittal **(G)** CT images of head displayed in a bone window reveal a large osteolytic and -productive mass in the right mandibular area. Mild asymmetric widening of the periodontal space around the caudal premolar and rostral mandibular molar teeth is noted in this area. Histopathology confirmed a squamous cell carcinoma. L, left; R, right.

The most common osseous tumors occurring in the head of the horse are osteomas, ossifying fibroma (Figure 17) and fibrous dysplasia; all are benign in their behavior and originate from the intramembranous bones of the head, typically in foals from 2 months to 1 year of age (82). Other, more aggressive tumors such as osteosarcoma are less frequently reported in the head of horses and can cause destructive osteolysis of one or multiple skull bones. Frequently osseous tumors are diagnosed on radiographs and no additional imaging is performed. Similar to other head neoplasia, CT has been demonstrated to provide more information about the precise location and extent of these lesions (19, 58). Several of these tumors have similar imaging characteristics and additional sampling is required to classify the tumor type (Figures 17A–G).

#### Temporomandibular Joint Disease

Standard radiographs of the head permit only limited evaluation of the temporomandibular joints. Ultrasound of the central and lateral aspects of the temporomandibular joints can be easily performed due to its superficial location; however, the medial aspect of the temporomandibular joint is generally not accessible using ultrasound. CT allows complete evaluation of the temporomandibular joints including the articular surfaces, and can be used to detect fractures (Figure 14C), subluxation or luxation and degenerative joint disease. In a retrospective study evaluating horses that were considered asymptomatic for temporomandibular joint disease, anatomical variations were reported in 40% of horses and 29% of the temporomandibular joints. These variations included round hypoattenuating areas in the condyles of the mandibles, suspected to be cystic lesions of the bone, and mineral attenuating areas in the intra-articular disks consistent with dystrophic mineralization. As this was a retrospective study, the clinical significance of these findings was unknown (83, 84). Currently, a limited number of studies describe temporomandibular joint disease in horses, including non-septic and septic arthritis (85, 86) and degenerative joint disease (87). Temporomandibular joint disease can be clinically challenging to diagnose, and CT is very useful in the investigation of this disease due to its ability to evaluate the entire joint.

#### Hyoid Apparatus Abnormalities

The hyoid apparatus can be evaluated using radiography; however, the degree of overlap of the individual parts of the hyoid apparatus in combination with the location of the temporohyoid articulation limits its complete diagnostic assessment. The temporohyoid articulation is most readily evaluated using endoscopy allowing visualization of this region within the guttural pouches. Additional information regarding the hyoid apparatus that can be obtained using CT includes the presence of new bone formation, fractures, displacement and malformation (88) (Figure 14D). In older horses, with no clinical signs of temporohyoid osteoarthropathy, remodeling of the temporohyoid joint can be noted on CT. These changes are frequently bilateral and include alteration of the shape of the proximal aspect of the stylohyoid bone, rounding of the synostosis with the petrous temporal bone and periarticular osteophyte formation. These changes are milder but similar to those commonly noted in horses with clinical temporohyoid osteoarthropathy (89). A recent publication documents the high incidence of temporal bone fractures identified on CT in horses with temporohyoid osteoarthropathy. All of these fractures were oriented in a rostrodorsal to caudoventral orientation and were minimally displaced (16). Computed tomography can also aid differentiation between osseous remodeling of the temporohyoid joint and osseous remodeling secondary to middle ear disease.

#### External Ear Canal, Tympanic Membrane and Bullae

Disease associated with the auditory system is infrequently reported in the horse. Most frequently reported diseases involve the external ear canal or are reported in combination with stylohyoid arthropathy. The normal auditory apparatus of horses has been described using CT (90); however, it is important to remember that similar to other species, the differentiation of small structures such as the tympanic membrane and auditory ossicles can be limited using CT.

### Other Head CT Findings

#### Soft Tissue Tumors of the Equine Head Not Related to the Sinus System, Ethmoid Bone or Teeth

Soft tissue tumors of the equine head include a mixed group of benign and malignant neoplasias arising from various non-epithelial and extra-skeletal structures including lymphatic and adipose tissue, smooth and skeletal muscles, and tendon, cartilage, fibrous tissue and blood vessels. Overall, the prevalence of neoplasia in horses is rather rare, but the head is a more commonly affected location in the horse (56). Differentiating various soft tissue tumor types using CT is often challenging as they share similar imaging features; however, CT provides useful information in staging the lesion by evaluating the origin, extent and vascularity of these masses (91), facilitating planning for biopsy or surgical resection. CT examination of soft tissue masses of the head should include intravenous iodinated contrast medium administration unless there is a known contraindication such as allergy. CT provides information in regard to the involvement of osseous structures and the brain (92), demonstrates mineralization of the soft tissue, and allows evaluation of the pattern and extent of vascularization, which may aid differentiation of various soft tissue tumor types (15).

Some commonly occurring tumors of the skin, such as sarcoids, occurring around the eyelids, ears and at the junction of the mouth (93) are frequently biopsied and surgically removed without the requirement for any imaging. However, in advanced stages CT might provide additional information regarding the extent of the lesion to help planning of further treatment.

**Figure 18:**
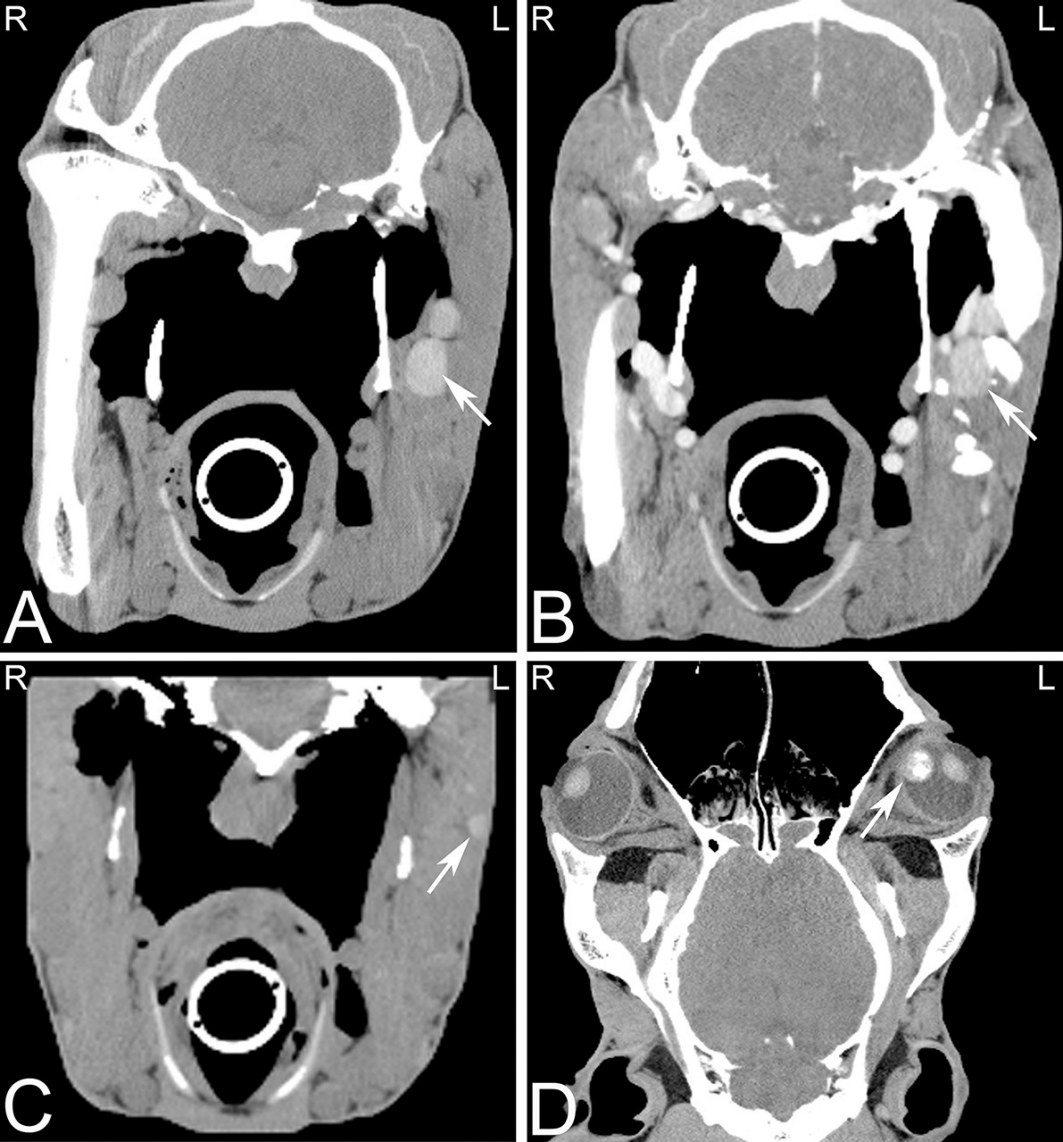
Multiple melanomas. Transverse **(A–C)** and dorsal **(D)** computed tomography images displayed in a soft tissue window of a 17-year-old Hanoverian horse demonstrating multiple hyperattenuating nodules in the musculature, pharyngeal and guttural pouch areas, and eye secondary to melanoma. CT images at the level of the guttural pouch before **(A)** and after intravenous iodinated contrast agent injection **(B)**. A round to ovoid uniform hyperattenuating group of nodules, which are mildly uniformly contrast enhancing is noted at the lateral aspect of the left guttural pouch. Additional hyperattenuating nodules were noted in musculature of the head **(C)** and in the medial aspect of the vitreous body of the left eye **(D)**. The nodule in the left eye showed also an area of heterogeneous mineral attenuation. L, left; R, right.

Melanomas are a common neoplasm in horses, and have been reported to involve the parotid salivary glands, skin, guttural pouches, paranasal sinuses and mucous membranes of the head or the melanin producing cells of the eye. Melanomas in horses are often initially slow growing and are most frequently noted in gray or white horses with black skin. Melanomas can be hyperattenuating compared to the musculature (Figure 18), which is reported to relate to the presence of intracytoplasmic melanin (94). However, melanomas in human patients have been reported to have a wide range of CT appearances (95) and it has been suggested that the hyperattenuation may also be secondary to hemorrhage within the mass. Most equine melanomas are benign and malignancy and metastasis to regional lymph nodes or distant organs can occur (Figures 18A–C).

**Figure 19:**
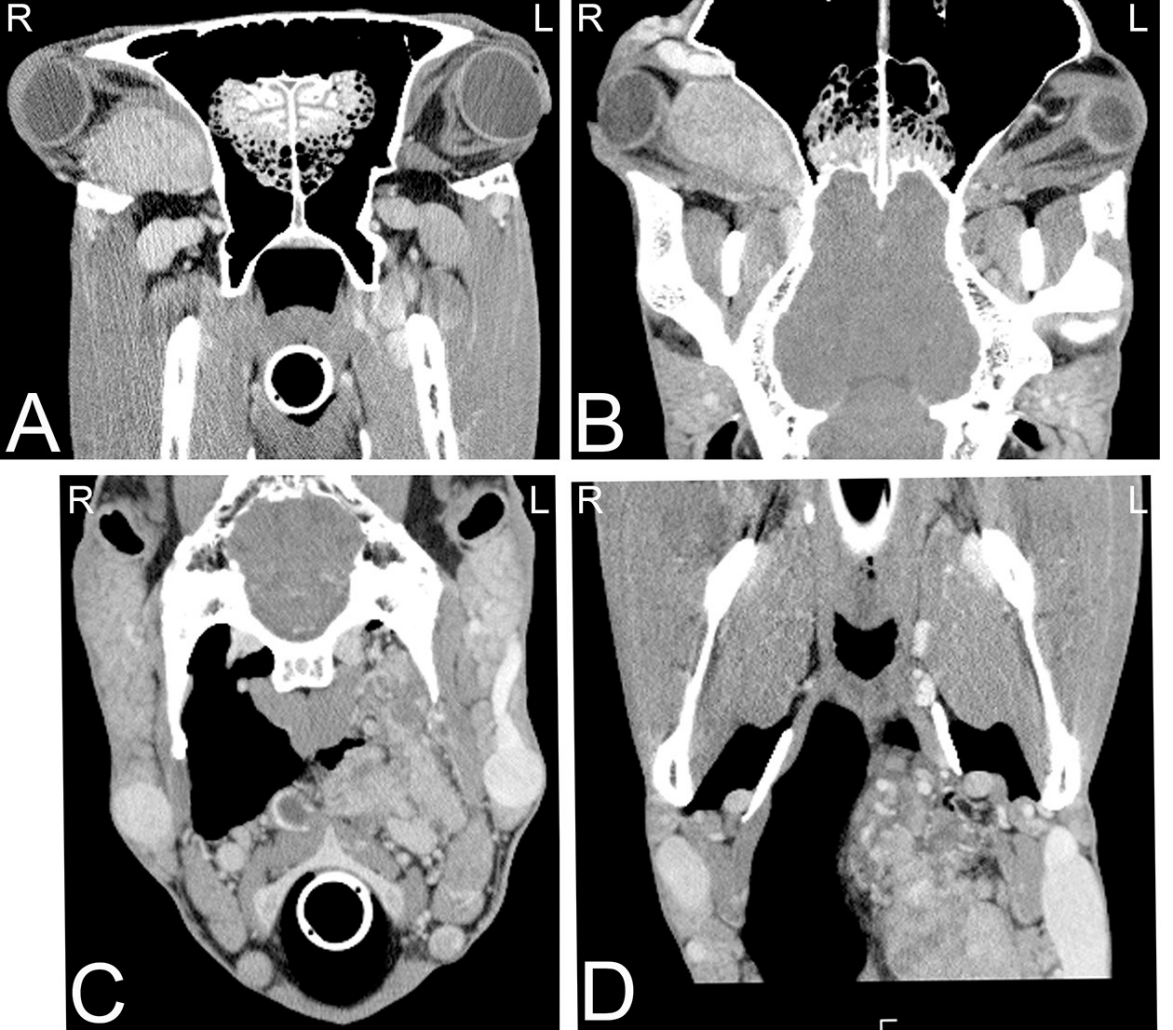
Neuroendocrine tumor. Transverse **(A,C)** and dorsal **(B,D)** computed tomography images post intravenous iodinated contrast medium injection displayed in soft tissue window demonstrating large retrobulbar **(A,B)** and retropharyngeal **(C,D)** masses, which were confirmed as a multicentric neuroendocrine tumors. CT images at the level of retrobulbar space **(A,B)** illustrate a large, well defined, mildly heterogeneously contrast enhancing mass, which displaces the eye laterally and caudally and the optical nerve caudally and does not involve the optical nerve. Additionally, a large lobulated, highly vascularized, moderately heterogeneously contrast enhancing mass is noted in the left retropharyngeal area **(C,D)**, resulting a reduced size of the left guttural pouch. L, left; R, right.

Squamous cell carcinoma also commonly affects the equine head, (Figures 17E–G), most frequently the oral cavity, involving the hard palate or pharynx. Similarly, lymphosarcoma commonly affects the head, and can occur in similar locations to squamous cell carcinomas (72). Rarely, neuroendocrine tumors are reported, usually in the retrobulbar region (Figure 19) causing exophthalmos (96). These tumors often extend through the cribriform plate into the neurocranium and in these cases, CT can provide critical information establishing a prognosis and treatment plan for these patients (19). Frequently when orbital tumors are diagnosed in horses, invasion of adjacent tissues has already occurred (97) and therefore advanced imaging is crucial for staging and treatment planning.

#### Sialocele or Sialadentitis

The horse has three distinct paired salivary glands including the parotid, mandibular (or submaxillary) and submandibular glands. The parotid gland is the largest and most frequently diseased salivary gland of the horse. Disease of the salivary gland can arise from ascending infection from the oral cavity, sialoliths, or from trauma to the head and neck. Parotid eoplasia has been reported but it very uncommon. Idiopathic bilateral swelling of the parotid gland (idiopathic parotitis or “grass glands”) commonly occurs and is not significant. Ultrasonic imaging represents a good choice of first line imaging. Evaluation of the salivary glands by CT is also possible and provides information about abnormal structures within or adjacent to the salivary gland and may identify the presence of dilated salivary ducts. Contrast enhanced CT results in enhancement of the salivary gland compared to adjacent tissue. Additionally, retrograde sialography can be performed to identify salivary duct obstruction, if indicated. Furthermore, mineral attenuating structures (sialoliths) may be identified in the salivary ducts. Currently, no CT studies are reported in horses focusing on the evaluation of the normal and diseased salivary glands.

#### Nasolacrimal Duct Obstruction

Nasolacrimal duct obstructions are uncommon in the horse but can occur secondary to a variety of etiologies (98). Indications for imaging the nasolacrimal duct include skull trauma or chronic epiphora possibly due to lacrimal bone suturitis. Computed tomography dacryocystography has been described in the horse and is a useful diagnostic tool in cases of persistent nasolacrimal duct obstruction (99).

## Conclusion and Clinical Relevance

Computed tomography is an excellent technique to evaluate the head in horses and can provide better anatomical understanding of a broad range of lesions. In addition, it has great value in the planning of interventional procedures and the removal of masses occurring on the head. CT may eliminate the need for other diagnostic imaging studies, especially when the CT can be performed in the standing patient. The ability to perform thin slices and multiplanar reconstructions of the head can help to more accurately evaluate head pathologies, especially in patients with trauma to the head, ear disease, or dental abnormalities.

## Author Contributions

SS-V contributed to conception and design of the manuscript and wrote the first draft of the manuscript. AH wrote sections of the manuscript. All authors contributed to manuscript revision, read, and approved the submitted version.

## Conflict of Interest

The authors declare that the research was conducted in the absence of any commercial or financial relationships that could be construed as a potential conflict of interest.

## Publisher's Note

All claims expressed in this article are solely those of the authors and do not necessarily represent those of their affiliated organizations, or those of the publisher, the editors and the reviewers. Any product that may be evaluated in this article, or claim that may be made by its manufacturer, is not guaranteed or endorsed by the publisher.
